# Continuous crystallization of pharmaceutical compounds in mixed suspension mixed product removal systems

**DOI:** 10.3389/fchem.2026.1947756

**Published:** 2026-10-16

**Authors:** Funeng Tan

**Affiliations:** Higher Vocational and Technical College, Wenshan University, Wenshan City, Yunnan Province, China

**Keywords:** continuous crystallization, MSMPR, pharmaceutical particle engineering, population-balance modeling, process analytical technology

## Abstract

**Background:**

Continuous pharmaceutical crystallization requires simultaneous control of supersaturation, particle attributes, polymorphic form, fouling, and downstream processability. Accordingly, single-stage and two-stage mixed-suspension mixed-product-removal (MSMPR) crystallization systems were developed and compared for the controlled production of the target crystalline polymorphs: paracetamol Form I and carbamazepine Form III.

**Methods:**

Equilibrium solubility and metastable-zone limits were determined before continuous operation. Four critical process parameters, residence time, relative supersaturation, continuous seed loading, and power input per unit volume, were evaluated using independent four-factor Box–Behnken designs. Response-surface optimization, stagewise population-balance modeling, multimodal process analytical technology, and matched configuration comparisons were performed at equal total active volume and overall residence time.

**Results:**

For both APIs, longer residence time increased median particle size toward the prespecified product-size ranges, narrowed particle-size distributions, and improved target-polymorph content, but reduced space-time yield. Higher supersaturation increased recovery and productivity for both APIs while broadening distributions and reducing polymorphic selectivity. Externally validated population-balance models achieved $R^{2}$ values of 0.894–0.943 and normalized RMSE values of 1.8%–8.8%. Relative to single-stage operation, two-stage MSMPR increased $D_{50}$ by 15.8% for paracetamol and 13.6% for carbamazepine, reduced PSD span by 14.6% and 16.9%, and decreased fouling by 42.4% and 45.8%, respectively. The larger, narrower particles were beneficial because filtration time declined by 26.3% for paracetamol and 34.4% for carbamazepine, while chemical purity and mass-balance closure were preserved.

**Conclusion:**

At matched active volume and overall residence time, staged supersaturation generation suppressed fines, improved particle uniformity and morphology, and reduced fouling and filtration time without compromising chemical or solid-state quality. These results identify controlled supersaturation distribution, not additional crystallizer capacity, as the principal benefit of two-stage MSMPR operation for the two APIs studied.

## 1 Introduction

Crystallization is a critical pharmaceutical unit operation because it determines active pharmaceutical ingredient purity, solid-form identity, particle-size distribution, and crystal morphology. These attributes directly affect filtration rate and time, drying, powder handling, and final product consistency. Batch crystallization remains widely used, but cyclic operation, scale-dependent mixing and heat transfer, batch-to-batch variability, and limited real-time intervention have encouraged continuous processing. Continuous crystallization can provide steady-state production, smaller equipment footprints, process intensification, and more consistent particle attributes. Realizing these benefits requires control of supersaturation, nucleation, growth, polymorphic transformation, residence-time distribution, mixing, withdrawal, fouling, and downstream solid–liquid separation (Capellades et al., 2018; Wood et al., 2019; Acevedo et al., 2019).

Continuous pharmaceutical crystallizers are commonly classified as tubular or plug-flow systems and mixed-suspension mixed-product-removal (MSMPR) systems. Tubular crystallizers offer narrow residence-time distributions and high surface-area-to-volume ratios, making them suitable for rapid cooling or antisolvent crystallization. However, they may be limited by wall deposition, blockage, solids transport, and sensitivity to particle loading. MSMPR crystallizers provide more flexible slurry handling, seeding, feed control, and scale-up. However, their broad residence-time distributions and back-mixing can promote the generation of fines, variable crystal histories, and premature particle washout (Wood et al., 2019; Ma et al., 2020; Orehek et al., 2020).

Single-stage MSMPR systems are experimentally accessible and permit transparent steady-state mass balances, but they must accommodate supersaturation generation, nucleation, growth, polymorphic selection, and desupersaturation within one vessel. These mechanisms often require conflicting conditions. High supersaturation can increase recovery and space-time yield but may broaden the particle-size distribution, intensify nucleation, worsen morphology, and reduce solid-form selectivity. Conversely, longer residence time and lower supersaturation may favor crystal growth and product quality while decreasing throughput (Ma et al., 2020; Orehek et al., 2020; Pascual et al., 2022; Pons-Siepermann et al., 2022). Multistage MSMPR configurations can alleviate this conflict by distributing cooling or antisolvent addition across vessels, thereby separating nucleation- and growth-dominated environments and providing additional control over particle size, morphology, and polymorphism (Capellades et al., 2018; Amari et al., 2022). Nevertheless, previous multistage studies have frequently focused on a single compound or have not compared configurations at matched total volume and overall residence time, making it difficult to isolate the true effect of staging (Capellades et al., 2018; Orehek et al., 2020; Amari et al., 2022).

Quality-by-design, response-surface methodology, and population-balance modeling have increasingly been used to define continuous-crystallization operating spaces. Statistical models efficiently identify interactions among residence time, supersaturation, seed loading, solvent composition, and agitation, but generally provide limited mechanistic interpretation outside the experimental domain. Population-balance models describe distributed crystal populations through nucleation, growth, withdrawal, agglomeration, breakage, and interstage transfer. However, their credibility requires parameter-identifiability assessment, uncertainty analysis, and independent validation rather than calibration performance alone (Pascual et al., 2022; Szilagyi et al., 2020; Barhate et al., 2024). Combining structured experimentation with mechanistic modeling therefore provides a stronger basis for optimization and configuration comparison.

Process analytical technology further enables real-time monitoring of concentration, particle evolution, morphology, and solid form. ATR-FTIR and Raman spectroscopy can estimate dissolved concentration and polymorphic content, whereas focused-beam reflectance measurement (FBRM) and image analysis provide complementary information on chord-length distributions and particle morphology. FBRM is a trend-sensitive chord-length method rather than a direct replacement for laser diffraction because its response depends on particle shape, orientation, and solids concentration (Capellades et al., 2018; Orehek et al., 2020; Domokos et al., 2021; Pal et al., 2024; Zhang et al., 2024). Comparatively few continuous-crystallization studies have validated several online measurements against independent offline references within one platform.

Operational robustness and downstream processability remain less extensively studied than yield and particle size. Fouling can alter heat transfer, mixing, vessel volume, residence-time distribution, PAT reliability, and mass-balance closure. At the same time, fines and elongated particles can increase cake resistance, filtration time, product loss, and mother-liquor retention (Wood et al., 2019; Acevedo et al., 2019; Aprile et al., 2025). Directly linking stagewise supersaturation control to fouling, filtration, morphology, and extended steady-state stability is therefore necessary for assessing manufacturing relevance.

The present study addresses these gaps using paracetamol Form I and carbamazepine Form III as contrasting model systems for cooling crystallization and polymorphic antisolvent-assisted cooling crystallization, respectively. Its specific scientific advance is a matched configuration comparison at identical total active volume and overall residence time across two APIs, combined with external population-balance validation, multimodal PAT validation, and simultaneous assessment of fouling and downstream filtration. This combination separates the effect of staging from added capacity and extends earlier single-compound or unmatched comparisons (Capellades et al., 2018; Orehek et al., 2020; Amari et al., 2022; Barhate et al., 2024). The investigation further evaluates product loss, cake resistance, process variability, mass-balance closure, and extended polymorphic stability, thereby connecting crystal engineering with operational and downstream performance. Contributions of the present study:

- A matched, compound-specific experimental and modeling workflow was implemented for two APIs with contrasting nucleation, morphology, and polymorphic behavior; it is presented as a reproducible demonstration rather than a universal framework.
- Residence time, relative supersaturation, seed loading, and power input were optimized through independent four-factor Box–Behnken designs.
- Stagewise population-balance models were calibrated, uncertainty quantified, and independently validated using particle-size, dissolved-concentration, and crystal-flow data.
- Single-stage and two-stage MSMPR systems were compared at matched total active volume and overall residence time.
- Multimodal PAT was validated against HPLC, quantitative XRPD, laser diffraction, and offline image analysis.
- Particle engineering was directly linked to fouling, filtration, cake resistance, product loss, and extended operational robustness.

## 2 Materials and methods

### 2.1 Study design and quality-by-design framework

This laboratory-scale original research study was designed to develop, optimize, and validate continuous crystallization of pharmaceutical compounds in mixed-suspension mixed-product-removal (MSMPR) systems. Two model active pharmaceutical ingredients (APIs) were selected to represent distinct crystallization challenges. Paracetamol was used as a rapidly nucleating compound suitable for cooling crystallization, whereas carbamazepine was used as a polymorphic and morphology-sensitive compound suitable for antisolvent-assisted cooling crystallization. The investigation combined equilibrium and metastable-zone characterization, hydrodynamic qualification, statistically designed single-stage MSMPR experiments, mechanistic population-balance modeling, and confirmatory assessment in a two-stage MSMPR cascade.

A quality-by-design approach (International Council for Harmonisation, 2009) was applied to link the candidate critical process parameters (CPPs) to the quality target product profile (QTPP), critical quality attributes (CQAs), and process-performance attributes. A preliminary failure-mode-and-effects assessment scored severity, occurrence, and detectability from 1 (low) to 5 (high); their product gave the risk-priority number (RPN), classified as high (≥40), moderate (20–39), or low (<20). The CQAs were median particle size, PSD span, aspect ratio, chemical purity, target-polymorph content, water content, and residual solvent. Recovery, space-time yield, filterability, fouling, mass-balance closure, and operating continuity were classified as process-performance attributes rather than CQAs. Table 1 states the corresponding targets and the risk-based links to residence time, relative supersaturation, continuous seed loading, and power input per unit volume.

**TABLE 1:** Quality target product profile, classification of quality and process-performance attributes, and risk-based linkage to candidate critical process parameters.

QTPP element	Class	Paracetamol target	Carbamazepine target	Linked CPPs	Initial RPN	Control/verification
Target polymorph	CQA	Form I ≥ 98.0%	Form III ≥98.0%	σ , seed loading, τP/V	48 (High)	Quantitative XRPD; DSC confirmation
Chemical purity		≥99.0% HPLC area	≥99.0% HPLC area	στ	36 (Moderate)	Validated HPLC
Median particle size $D_{50}$		220–280 µm	150–190 µm	τ , σ , seed loading P/V	48 (High)	Laser diffraction
PSD span		≤1.30	≤1.50	τ , σ , seed loading P/V		$D_{10}$ , $D_{50}$ , and $D_{90}$ by laser diffraction
Particle morphology		Aspect ratio ≤1.70	Aspect ratio ≤4.00	σ , seed loading P/V	36 (Moderate)	SEM and calibrated image analysis (≥200 particles)
Water/residual solvent		Water ≤0.50%	Water ≤0.50%; ethanol ≤0.50%	Drying temperature/time; solvent composition	24 (Moderate)	Karl Fischer and headspace GC
Isolated recovery	Process-performance attribute	≥18.0%	≥18.0%	τ , σ , seed loading P/V		Dry crystal mass flow and liquid-phase balance
Space-time yield		≥15.0 g L^‒1^ h^‒1^	≥5.0 g L^‒1^ h^‒1^	τ , σP/V		Dry crystal mass flow per active volume
Filterability		≤45 s per 100 mL	≤75 s per 100 mL	PSD span, morphology, fines	27 (Moderate)	Constant-pressure filtration
Operational robustness		Fouling ≤0.75 g m^‒2^ h^‒1^; no interruptions	Fouling ≤0.90 g m^‒2^ h^‒1^; no interruptions	σ , P/V , withdrawal, RTD	36 (Moderate)	Deposits, flow stability, PAT signals; mass-balance closure 95%–105%

### 2.2 Materials and model compounds

Paracetamol (pharmaceutical secondary-standard grade, assay 99.7%; Sigma-Aldrich, St. Louis, MO, United States) and carbamazepine (pharmaceutical-analysis grade, assay 99.6%; Tokyo Chemical Industry, Tokyo, Japan) were used without further chemical purification. HPLC-grade ethanol (≥99.9%), acetonitrile (≥99.9%), and methanol (≥99.9%), and LC–MS-grade formic acid (≥99.0%) were supplied by Merck/Supelco (Darmstadt, Germany). Ultrapure water was prepared with a Milli-Q IQ 7000 system (Merck Millipore, Burlington, MA, United States) at 18.2 MΩ·cm and ≤5 ppb total organic carbon. Hydrophilic PTFE syringe filters (Millex, 25-mm devices; 0.45-µm and 0.22-µm pore sizes; Merck Millipore, Burlington, MA, United States) were used for solvent and undersaturated API-feed filtration, respectively. Supplier, catalog and batch numbers, assay, water content, and certificate-of-analysis information were recorded for every material.

Paracetamol Form I was crystallized from water by controlled cooling. Carbamazepine Form III was crystallized from an ethanol–water system by antisolvent addition and moderate cooling. The QTPP was established before the designed experiments; Table 1 separates product-quality targets from process-performance targets and reports the initial CPP-to-CQA risk assessment.

### 2.3 Initial solid-state and physicochemical characterization

The received APIs and prepared seeds were characterized before continuous processing. XRPD used a D8 Advance diffractometer (Bruker, Karlsruhe, Germany) with Cu Kα radiation at 40 kV and 40 mA; patterns were collected from 3° to 40° in 2θ at 0.02° steps and 0.5 s per step. DSC used a DSC 250 instrument (TA Instruments, New Castle, DE, United States) under nitrogen at 50 mL min^‒1^ with 3.0–5.0 mg samples heated at 10 °C min^‒1^. Thermogravimetric analysis used a Discovery TGA 550 instrument (TA Instruments) from 25 °C to 300 °C at 10 °C min^‒1^ under nitrogen. SEM images were acquired with a Sigma 300 VP microscope (Carl Zeiss, Oberkochen, Germany) at 5 kV after applying an 8-nm gold coating. At least 200 particles per sample were analyzed for length, width, equivalent circular diameter, and aspect ratio in ImageJ 1.54g (National Institutes of Health, Bethesda, MD, United States).

Particle-size distributions were measured in triplicate with a Mastersizer 3000 equipped with a Hydro MV wet-dispersion unit (Malvern Panalytical, Malvern, United Kingdom), using Mie theory, 2,000 rpm stirring, and 10%–15% obscuration in silicone oil (10 cSt; Sigma-Aldrich, St. Louis, MO, United States) as the non-dissolving dispersant; the refractive index of the dispersant was set to 1.403 at 633 nm, with particle refractive indices of 1.58 for paracetamol and 1.60 for carbamazepine. Chemical identity, purity, and dissolved concentration were determined with a Nexera X2 HPLC system and SPD-M30A photodiode-array detector (Shimadzu, Kyoto, Japan). Water content was measured with an 899 Coulometer (Metrohm, Herisau, Switzerland) using Hydranal Coulomat AG reagent (Honeywell Fluka, Seelze, Germany). Residual solvents were measured with an 8890 gas chromatograph and 7697A headspace sampler (Agilent Technologies, Santa Clara, CA, United States) equipped with a DB-624 UI column (30 m × 0.32 mm × 1.8 µm; Agilent J&W); helium flow was 1.5 mL min^‒1^, headspace equilibration was 20 min at 80 °C, the oven was held at 40 °C for 5 min and ramped at 10 °C min^‒1^–180 °C, and the FID was maintained at 250 °C.

### 2.4 Equilibrium solubility and metastable-zone determination

Equilibrium solubility was determined by the isothermal shake-flask method. Excess API was added to sealed vessels containing the selected solvent or solvent-antisolvent composition. Suspensions were agitated at 300 rpm for 24 h and then maintained for a further 24 h at the target temperature. Preliminary tests verified that the dissolved concentration did not change materially between 24 and 48 h. Paracetamol solubility was measured in water at 10, 15, 20, 25, 30, 40, and 50 °C. Carbamazepine solubility was measured at 15, 20, and 25 °C in ethanol-water mixtures containing 0%–60% water by volume. Samples were withdrawn using temperature-conditioned syringes, filtered immediately through 0.22-µm membranes, diluted with the HPLC mobile phase, and analyzed in triplicate. The supersaturation ratio, S , and relative supersaturation, σ , were calculated from the measured dissolved concentration, c , and the equilibrium concentration, $c_{\text{eq}}$ , at the corresponding temperature and solvent composition (Equations 1,2):

$$S=\frac{c}{c_{\text{eq}}}$$ (1)

$$\sigma =S-1=\frac{c-c_{\text{eq}}}{c_{\text{eq}}}$$ (2)

Metastable-zone limits were determined in a 100-mL jacketed glass vessel fitted with an IKA Eurostar 60 digital overhead stirrer (IKA, Staufen, Germany), a calibrated Pt100 probe (±0.1 °C), a ParticleTrack G400 FBRM probe, and a ParticleView V19 microscope (both Mettler Toledo, Columbus, OH, United States). FBRM data were acquired over 1–1,000 µm at 2-s intervals. Solutions were heated until dissolution and held for 20 min to erase thermal history, then cooled at exactly 0.20, 0.50, or 1.00 °C min^‒1^. The nucleation point was the temperature at which total FBRM counts exceeded the baseline by five standard deviations for at least 30 s and particles were confirmed by microscopy. Each condition was repeated three times. The continuous operating range was restricted below the experimentally measured uncontrolled-nucleation boundary.

### 2.5 Seed preparation and qualification

Seeds of the target polymorph were produced by slow batch crystallization under conditions favoring the intended solid form. Recovered crystals were vacuum filtered, washed with cold API-saturated solvent, and dried under vacuum at 40 °C to constant mass. The dried crystals were gently milled and sieved, and the 75–125 µm fraction was retained. Seed identity was verified by XRPD and the seed-size distribution was confirmed by laser diffraction. For continuous dosing, seeds were suspended in API-saturated mother liquor maintained at the operating temperature. The seed reservoir was continuously agitated to prevent settling, and the slurry was delivered through wide-bore tubing using a calibrated pump. Continuous seed loading, $\phi_{\text{seed}}$ , was defined relative to the dissolved API mass flow entering the crystallizer (Equation 3):

$$\phi_{\text{seed}}=100\;\frac{F_{\text{seed}}}{F_{\text{API},\text{feed}}}$$ (3)

where $F_{\text{seed}}$ is the seed mass-flow rate and $F_{\text{API},\text{feed}}$ is the dissolved API mass-flow rate.

### 2.6 MSMPR crystallization platform

The experimental platform used modular borosilicate jacketed MSMPR vessels, an IKA Eurostar 60 digital overhead stirrer, CORIO CD-1000F recirculating thermostatic baths (JULABO, Seelbach, Germany), and Watson-Marlow 120U/DV calibrated feed and withdrawal pumps (Watson-Marlow, Falmouth, United Kingdom). Single-stage experiments used a 150-mL working volume. Two-stage experiments used two geometrically similar 75-mL vessels in series, preserving the same total active volume. Each vessel contained a four-blade pitched-blade turbine positioned at 1.0 impeller diameter above the vessel base. Baffles suppressed vortex formation. The withdrawal dip tube was placed in the well-mixed region and sized to pass the upper PSD tail without classification or blockage. Temperature was measured with calibrated Pt100 probes with an accuracy of ±0.1 °C.

Feed and outlet pumps were calibrated gravimetrically before each experimental block. A calibration was accepted when the measured flow differed from the set point by less than 2%. Feed reservoirs and transfer lines were maintained under conditions that prevented premature crystallization. The paracetamol feed line was heated and insulated, whereas carbamazepine solution and water antisolvent were introduced through separate inlets located within the impeller-discharge region. The nominal mean residence time, τ , was calculated from the active vessel volume, V , and the total volumetric flow rate, $Q_{T}$ (Equation 4):

$$\tau =\frac{V}{Q_{T}}$$ (4)

For the two-stage cascade, the total nominal residence time was the sum of the stage residence times. The total active volume and overall residence time were matched to those of the corresponding single-stage experiment to permit a direct configuration comparison. The overall experimental platform and workflow used to compare the single-stage and two-stage MSMPR configurations are summarized in Figure 1.

**FIGURE 1:**
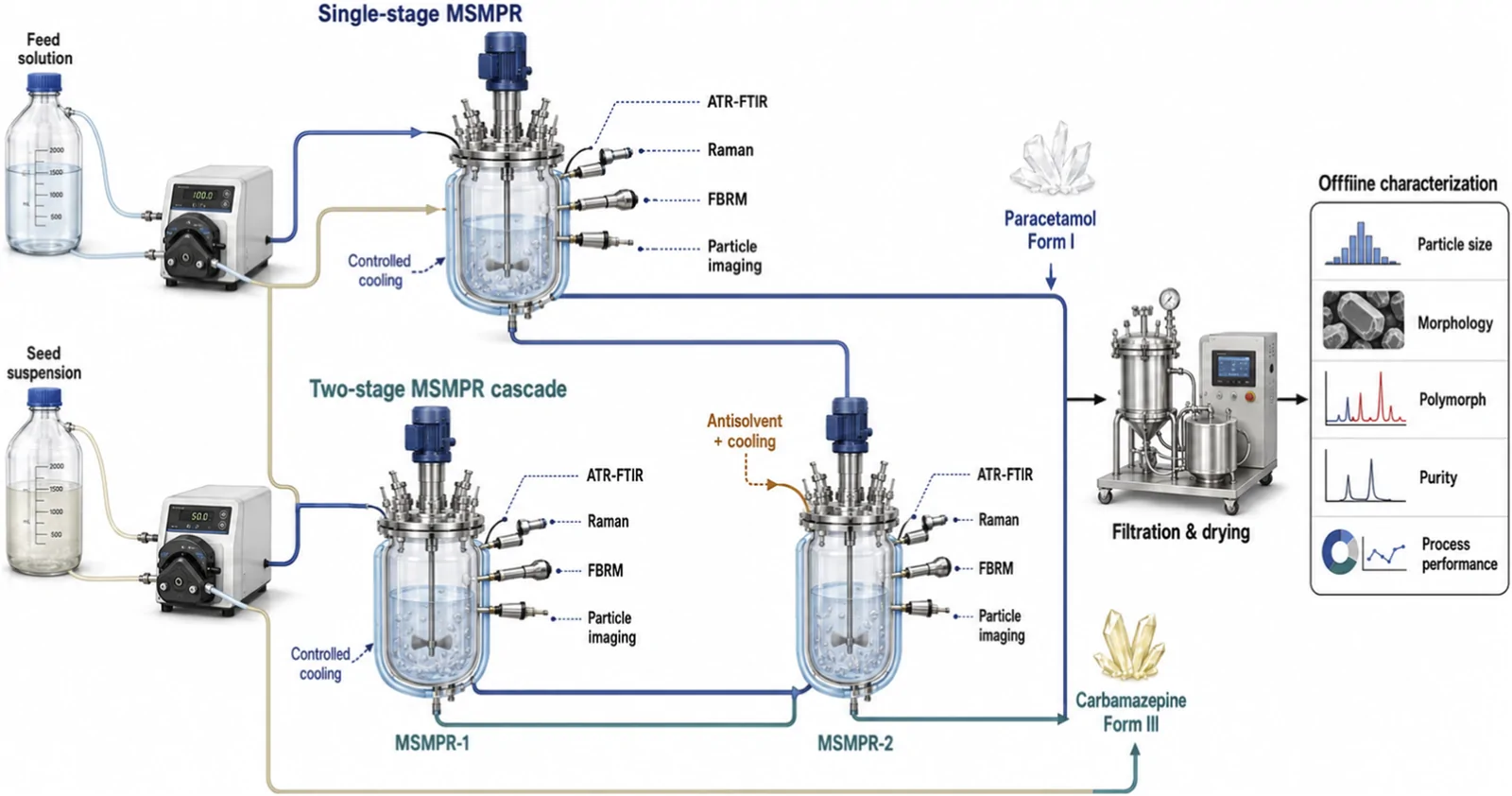
Schematic of the single- and two-stage MSMPR crystallization platform for paracetamol Form I and carbamazepine Form III, including process monitoring, product isolation, and characterization.

### 2.7 Hydrodynamic and residence-time qualification

The just-suspended impeller speed was established using representative crystals. Experimental agitation rates were maintained at least 15% above this value to prevent persistent settling. Liquid-phase mixing time was measured after pulse addition of a conductivity tracer. The mixing time, $t_{95}$ , was defined as the interval required for the conductivity signal to remain within ±5% of its final value. The selected agitation condition was required to satisfy $t_{95}\leq 0.10\tau$ . Residence-time distribution (RTD) experiments were performed before API crystallization. A short pulse of a non-interacting conductivity tracer was introduced into the inlet, and outlet conductivity was recorded at 1-s intervals. Solid-phase RTD was assessed using size-matched fluorescent or optically distinguishable tracer particles with density approximating that of the API crystals. The normalized exit-age distribution, $E_{t}$ , was calculated from the background-corrected tracer concentration, $C_{t}$ (Equation 5):

$$E_{t}=\frac{C_{t}}{\int_{0}^{\infty }C_{t}\;dt}$$ (5)

The experimental mean residence time, $\bar{t}$ , was obtained from the first moment of $E_{t}$ and was required to remain within 10% of the nominal value. RTD qualification was repeated at the lowest and highest flow rates and at a representative slurry concentration. Evidence of bypassing, dead volume, or persistent particle retention required modification of the dip-tube position, impeller speed, or vessel configuration before continuation.

### 2.8 Continuous crystallization procedures

#### 2.8.1 General start-up and material diversion

Before each run, the crystallizer and transfer lines were cleaned, inspected, rinsed with the corresponding solvent, and leak-tested. The vessel was initially charged with API-saturated mother liquor having the same temperature and solvent composition as the intended steady-state liquid phase. Agitation and temperature control were initiated, followed by addition of the initial seed bed. Once temperature stabilized within ±0.2 °C, the solution, antisolvent when applicable, seed-slurry, and product-withdrawal pumps were started simultaneously. Start-up material was diverted from the product stream. Formal steady-state sampling did not begin before five nominal residence times had elapsed and all predefined steady-state criteria had been satisfied.

#### 2.8.2 Paracetamol cooling crystallization

A paracetamol solution corresponding to 90% of the equilibrium solubility at 50 °C was prepared under agitation and maintained at 50 °C ± 0.5 °C. The feed was transferred through a heated line to the MSMPR vessel. Supersaturation was generated by cooling to the experimental set point corresponding to the assigned value of σ . Temperature, total flow, seed flow, and agitation were maintained at their prescribed levels until steady state was confirmed.

#### 2.8.3 Carbamazepine antisolvent-assisted crystallization

Carbamazepine was dissolved in ethanol at 25 °C to 85% of its equilibrium solubility. The API solution and water antisolvent were delivered through independent calibrated pumps. The antisolvent flow was selected using the experimentally determined solubility relationship to achieve the assigned value of σ at 15 °C. The two streams were introduced within the high-energy impeller-discharge region while preventing direct mixing inside the feed tubes. Carbamazepine Form III seed slurry was supplied continuously.

#### 2.8.4 Two-stage MSMPR operation

The statistically optimized operating condition for each API was subsequently tested in a two-stage cascade. For paracetamol, the total cooling duty was divided between stages to establish moderate supersaturation in stage 1 and complete the crystallization in stage 2. For carbamazepine, the total antisolvent flow was split between stages to avoid a single high-supersaturation pulse. The initial distribution assigned 60% of the supersaturation-generating change to stage 1% and 40% to stage 2. The split was refined using the mechanistic model while preserving the same total flow, active volume, and nominal overall residence time as the matched single-stage condition.

### 2.9 Design of experiments and optimization

A four-factor Box–Behnken response-surface design was constructed independently for each API. The coded factors were residence time (A; 15, 30, and 45 min), relative supersaturation (B; 0.10, 0.20, and 0.30), seed loading (C; 0.25%, 0.75%, and 1.25%), and power input per unit volume (D; 0.20, 0.45, and 0.70 W L^‒1^). Each design contained 24 edge combinations and five center-point runs, giving exactly 29 experiments per compound. Runs were blocked by day and randomized within block; center points were distributed through the campaign. Each response was fitted with the hierarchical second-order model (Equation 6):

$$ŷ=\beta_{0}+\overset{4}{\underset{i=1}{\Sigma }}\left(\beta_{i}x_{i}+\beta_{\text{ii}}x_{i}^{2}\right)+\Sigma_{i<j}\beta_{\text{ij}}x_{i}x_{j}$$ (6)

where ŷ is the predicted response; $\beta_{0}$ , βᵢ , βᵢᵢ , and βᵢj are the intercept, linear, quadratic, and interaction coefficients; and xᵢ and xj are coded factor levels. Model adequacy was evaluated by ANOVA, adjusted and predicted coefficients of determination, lack-of-fit testing, studentized residuals, influence diagnostics, and variance-inflation factors.

A multiresponse desirability function maximized $D_{50}$ within the QTPP ranges in Table 1, isolated recovery, space-time yield, chemical purity, and polymorphic purity, while minimizing PSD span, aspect ratio, supersaturation, and fouling. Conditions that failed chemical-purity or solid-form requirements were excluded from the feasible space. The optimum maximized overall desirability. The robustness condition was prespecified as a high-throughput boundary point within 5% of an upper coded level for supersaturation or power input, retaining at least 95% of the predicted maximum recovery and STY while meeting ≥99.0% chemical purity and ≥98.0% target-polymorph content. Each condition was evaluated in three independent runs.

All 58 Box–Behnken experiments and the results reported in Sections 3.2 and 3.3 used the 150-mL single-stage configuration. The two-stage cascade was introduced only for the matched confirmatory comparison reported in Section 3.5.

### 2.10 Process analytical technology and steady-state definition

Each crystallizer was monitored with calibrated Pt100 temperature probes, the ParticleTrack G400 FBRM probe, and the ParticleView V19 microscope. Paracetamol concentration was monitored with a ReactIR 702L ATR-FTIR system and DiComp probe (Mettler Toledo) at 4 cm^‒1^ resolution using 128 co-added scans per spectrum. Carbamazepine concentration and Form III content were monitored with a RamanRXN2 analyzer (Kaiser Optical Systems, Ann Arbor, MI, United States) using a 785-nm laser at 400 mW, three 5-s accumulations, and a 150–1,800 cm^‒1^ spectral range. FBRM chord counts were reported in <10, 10–50, 50–150, and >150 µm channels and were interpreted as trends rather than laser-diffraction-equivalent sizes.

Compound-specific partial-least-squares models used calibration samples spanning the complete concentration, temperature, and solvent-composition domains. Spectra were pretreated by Savitzky–Golay first derivatives (second-order polynomial, 11-point window) and mean centering. Latent variables were selected by sevenfold cross-validation. A calibration was accepted when independent-validation error was <5% of the concentration range and no concentration-dependent bias was detected.

### 2.11 Sampling, isolation, and product characterization

Slurry was collected through the product outlet without interrupting continuous flow; each sample represented at least 2 min of outlet flow. A known slurry mass was divided immediately into liquid and solid fractions. Dissolved concentration was measured after rapid filtration through a preconditioned membrane. Solids were vacuum filtered through a 47-mm glass-fiber filter (Whatman GF/C, nominal retention 1.2 µm; Cytiva, Maidstone, United Kingdom) selected to retain the fines fraction, washed with minimal cold API-saturated solvent, and dried at 40 °C until two measurements separated by 2 h differed by <0.2%. Chemical purity and dissolved concentration were measured on the Shimadzu Nexera X2 system using a Shim-pack GIST C18 column (150 × 4.6 mm, 5 μm; Shimadzu GLC, Tokyo, Japan) at 30 °C, a 1.0 mL min^‒1^ flow, and 10-µL injection. Paracetamol used water/acetonitrile/formic acid (80:20:0.1, v/v/v) at 243 nm; carbamazepine used water/acetonitrile/formic acid (35:65:0.1, v/v/v) at 285 nm. Six-level calibrations covered 50%–150% of sample concentration and required $R^{2}$ >0.999. System suitability required peak-area RSD ≤2.0%, tailing factor 0.8–1.5, ≥5,000 theoretical plates, and resolution ≥2.0. Laser diffraction results were reported as $D_{10}$ , $D_{50}$ , and $D_{90}$ ; PSD width was calculated as follows (Equation 7):

$$W_{\text{PSD}}=\frac{D_{90}-D_{10}}{D_{50}}$$ (7)

Morphology was evaluated by scanning electron microscopy and particle-video microscopy. At least 200 particles per condition were analyzed for aspect ratio. Polymorphic identity and purity were determined by quantitative XRPD using whole-pattern fitting or Rietveld refinement and were supported by differential scanning calorimetry. Water content and residual solvent were measured by Karl Fischer titration and headspace gas chromatography, respectively. Filterability was assessed by constant-pressure filtration using a fixed slurry mass and filter area.

### 2.12 Material balance and process-performance calculations

The dissolved API mass entering the system was calculated from the sum of the API-containing inlet streams. The API leaving the system was represented by dissolved API in the mother liquor and isolated crystalline product. Mass-balance closure, $C_{\text{MB}}$ , was calculated as (Equation 8):

$$C_{\text{MB}}\;\left(\%\right)=100\;\frac{F_{\text{API},\text{out}}}{F_{\text{API},\text{feed}}}$$ (8)

where $F_{\text{API},\text{feed}}$ and $F_{\text{API},\text{out}}$ are the total API mass-flow rates entering and leaving the system, respectively. Isolated recovery, $R_{\text{iso}}$ , and space-time yield, $Y_{\text{ST}}$ , were calculated from the dry crystal mass-flow rate, $F_{\text{crystal}}$ , and the total active crystallizer volume, $V_{\text{total}}$ (Equations 9,10):

$$R_{\text{iso}}\;\left(\%\right)=100\;\frac{F_{\text{crystal}}}{F_{\text{API},\text{feed}}}$$ (9)

$$Y_{\text{ST}}=\frac{F_{\text{crystal}}}{V_{\text{total}}}$$ (10)

The difference between recovery estimated from mother-liquor depletion and recovery measured from isolated dry product was used to detect unmeasured fines, sampling losses, deposits, or analytical bias.

### 2.13 Population-balance and kinetic modeling

A stagewise, one-dimensional population-balance model was developed to describe nucleation, crystal growth, withdrawal, and interstage transfer. Agglomeration or breakage terms were incorporated only when microscopy, particle-size residuals, and model-selection criteria demonstrated that they were necessary. For stage i , the crystal population density, $n_{i}$ , was described by (Equation 11):

$$\frac{\partial n_{i}}{\partial t}+\frac{\partial \left(G_{i}n_{i}\right)}{\partial L}=B_{0,i}\delta \left(L-L_{0}\right)+\frac{n_{i-1}-n_{i}}{\tau_{i}}$$ (11)

Where L is characteristic crystal size, $G_{i}$ is the linear growth rate, $B_{0,i}$ is the nucleation rate, $L_{0}$ is the nucleus size, δ is the Dirac delta function, and $\tau_{i}$ is the residence time of stage i . For the first stage, $n_{i-1}$ represented the continuously supplied seed population. Growth and nucleation kinetics were initially represented by power-law expressions (Equations 12,13):

$$G_{i}=k_{g}\sigma_{i}^{g}$$ (12)

$$B_{0,i}=k_{b}\sigma_{i}^{b}M_{T,i}^{j}$$ (13)

where $k_{g}$ and $k_{b}$ are kinetic constants, g and b are supersaturation exponents, $M_{T,i}$ is suspension density, and j describes the dependence of secondary nucleation on the suspended crystal mass. The population-balance equation was coupled to a dissolved-phase mass balance. The equations were solved using a finite-volume method with grid- and time-step-independence verification. Numerical settings were accepted when predicted median size, upper-tail size, and suspension density changed by less than 1% after refinement.

Kinetic parameters were estimated simultaneously from steady-state dissolved concentrations, particle-size distributions, and dry crystal production rates. Parameter positivity was enforced by logarithmic transformation. A hierarchical modeling strategy was used: nucleation and growth were estimated first, followed by addition of agglomeration, breakage, or size-dependent growth only when systematic residuals remained. Parameter uncertainty was quantified using 1,000 bootstrap resamples, and practical identifiability was assessed from profile likelihoods and parameter-correlation matrices.

### 2.14 Model validation and configuration comparison

For each API, exactly 22 of the 29 designed experiments (75.9%) formed the calibration set and seven (24.1%) formed the internal-validation set, with both sets spanning the coded factor domain. All observations derived from one experimental run-including repeated analytical measurements from a center-point run, were assigned to one partition; no measurement from a calibration run reappeared in validation. Predictive performance was evaluated by root-mean-square error (RMSE), normalized root-mean-square error (NRMSE; RMSE divided by the observed response range and expressed as a percentage), mean absolute percentage error (MAPE), residual plots, and predicted-versus-measured particle distributions. Nine independent optimized, robustness, and two-stage experiments per API constituted external validation. Cascade predictions used the previously estimated kinetic parameters without refitting and only measured stage volumes, flows, temperatures, solvent compositions, seed conditions, and RTD characteristics. Configuration superiority required a practically meaningful gain in at least one primary quality or productivity response without violating chemical-purity, polymorphic-purity, mass-balance, or stability requirements.

### 2.15 Fouling and operational robustness

Fouling was monitored using process images, temperature-control behavior, outlet-flow stability, probe signal quality, and deposit recovery after each run. Wall and probe deposits were collected separately, dried, and weighed. Fouling rate, $r_{f}$ , was normalized by wetted surface area, $A_{\text{wetted}}$ , and continuous operating time, $t_{\text{run}}$ (Equation 14):

$$r_{f}=\frac{m_{\text{deposit}}}{A_{\text{wetted}}t_{\text{run}}}$$ (14)

An operational interruption was recorded when the flow deviated by more than 5%, the vessel volume deviated by more than 3%, a feed or outlet line required manual intervention, or a PAT probe became obscured by deposits. Optimized conditions were evaluated during extended operation for at least 12 additional nominal residence times after steady state to assess drift, encrustation, polymorphic stability, and outlet consistency.

### 2.16 Statistical analysis, reproducibility, and data integrity

Continuous variables were summarized as mean ± standard deviation when the Shapiro–Wilk test did not reject normality (p ≥ 0.05) and as median with interquartile range otherwise. Response-surface models were assessed by ANOVA, lack-of-fit tests, residual normality and homoscedasticity, influence diagnostics, and variance-inflation factors. Single-stage and two-stage configurations were compared with linear models containing compound, configuration, and their interaction. Run identity was a random effect when several steady-state samples were analyzed from one run. Pairwise comparisons used the Holm sequential procedure (Holm, 1979). All tests were two-sided at p < 0.05, and effect estimates are reported with 95% confidence intervals.

Each confirmatory condition was performed in three independent runs using independently prepared feed and seed suspensions. Analytical samples were coded before offline characterization when feasible. Pump calibrations, instrument checks, raw PAT signals, sampling records, processing scripts, deviations, and excluded observations were retained in an auditable electronic record. A run was excluded only for a documented equipment failure, incorrect material preparation, loss of temperature control, pump malfunction, or unacceptable mass-balance closure accompanied by an identifiable procedural cause. Failed runs were reported and repeated rather than silently removed.

Box–Behnken design, ANOVA, desirability optimization, and diagnostic plots were generated in Design-Expert 13.0.5 (Stat-Ease, Minneapolis, MN, United States). Population-balance equations were solved in MATLAB R2024b (MathWorks, Natick, MA, United States). Data curation and confirmatory statistics used Python 3.12.4 with NumPy 2.0.1, SciPy 1.14.0, pandas 2.2.2, statsmodels 0.14.2, and scikit-learn 1.5.1. Figures were assembled in MATLAB R2024b and Python Matplotlib 3.9.1.

### 2.17 Safety and waste management

Experiments involving organic solvents were performed in a ventilated enclosure using closed, grounded feed reservoirs and solvent-compatible tubing. Appropriate personal protective equipment was selected according to the safety data sheets of the APIs and solvents. API-containing solvent waste, aqueous cleaning waste, crystalline residues, and contaminated filters were segregated and disposed of according to institutional hazardous-waste procedures. No human participants, animals, human-derived specimens, or identifiable personal data were involved.

## 3 Results

### 3.1 Equilibrium solubility and metastable-zone characteristics

Paracetamol and carbamazepine showed distinct solubility and metastable-zone behavior. Figure 2A shows that paracetamol solubility in water increased 5.99-fold, from 8.82 ± 0.14 g L^‒1^ at 10 °C to 52.80 ± 0.69 g L^‒1^ at 50 °C, with low relative standard deviations of 1.31%–1.59%. As shown in Figure 2B, increasing the cooling rate from 0.20 °C to 1.00 °C min^‒1^ widened the metastable-zone width from 9.3 ± 0.5 to 15.1 °C ± 0.7 °C, lowered the nucleation temperature from 38.8 ± 0.4 to 33.0 °C ± 0.6 °C, and increased the relative supersaturation at nucleation from 0.508 to 1.048. For carbamazepine, Figure 2C demonstrates that increasing water content from 0% to 60% v/v at 15 °C reduced solubility by 91.3%, from 21.00 ± 0.28 to 1.82 ± 0.04 g L^‒1^, while higher temperature increased solubility at each solvent composition. Figure 2D shows that increasing the cooling rate from 0.20 °C to 1.00 °C min^‒1^ at 30% v/v water widened the metastable zone from 10.8 ± 0.5 to 16.6 °C ± 0.7 °C, reduced the nucleation temperature from 24.4 ± 0.4 to 18.6 °C ± 0.6 °C, and increased the relative supersaturation at nucleation from 0.383 to 0.634. Overall, these data defined the compound-specific operating boundaries for continuous crystallization.

**FIGURE 2:**
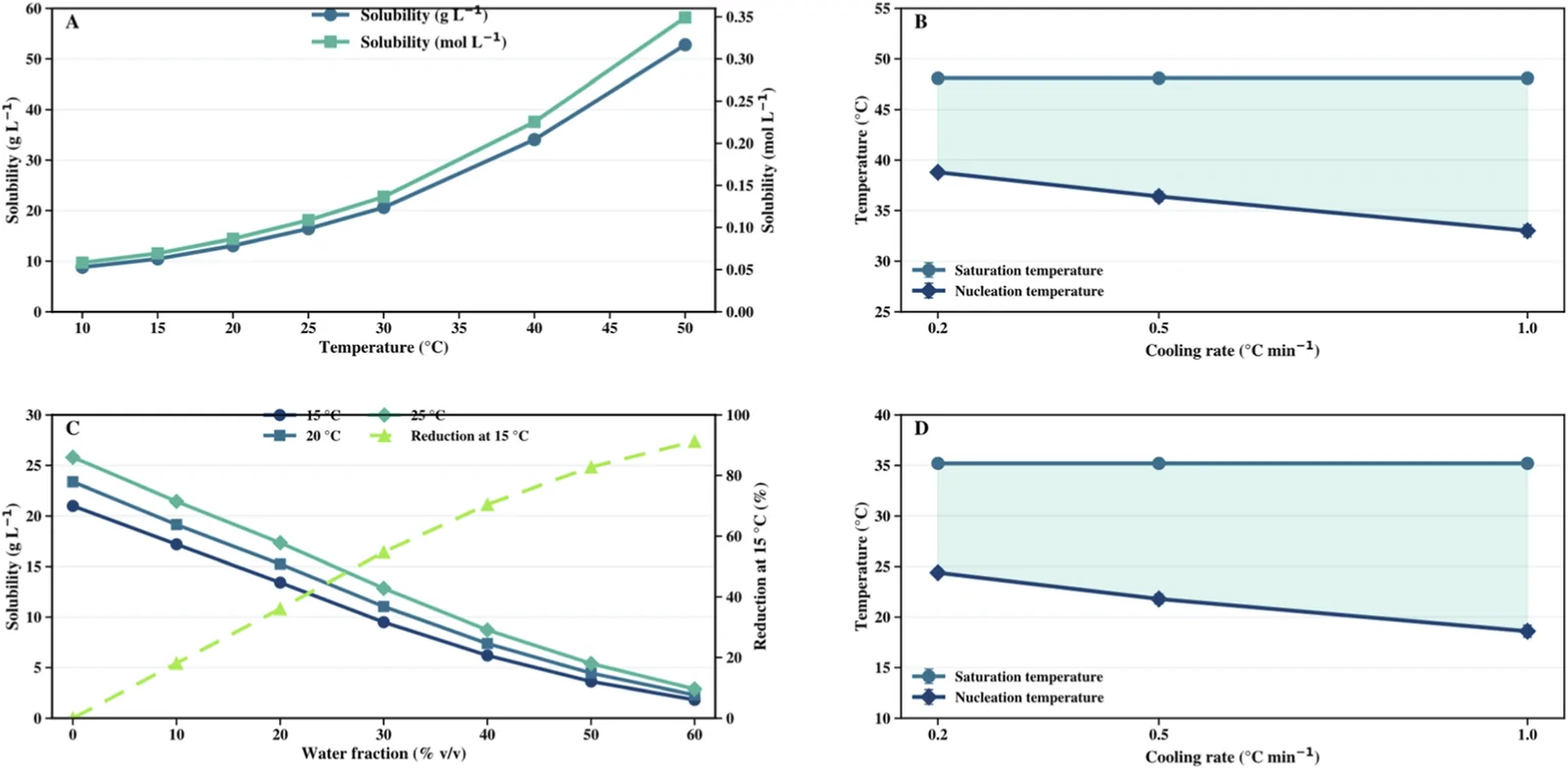
Solubility and metastable-zone characteristics of paracetamol and carbamazepine. **(A)** Paracetamol solubility versus temperature. **(B)** Cooling-rate effects on paracetamol nucleation. **(C)** Carbamazepine solubility versus water fraction and temperature. **(D)** Cooling-rate effects on carbamazepine nucleation at 30% v/v water. Data are mean ± SD (n = 3).

### 3.2 Effects of critical process parameters on product quality and process performance

All factor-effect estimates in Figure 3 were obtained with the 150-mL single-stage MSMPR. The critical process parameters produced consistent but compound-dependent changes in particle attributes. Figure 3A shows that increasing residence time from 15 to 45 min generated the largest increase in $D_{50}$ : 87.0 µm for paracetamol and 59.0 µm for carbamazepine (both p < 0.001). Increasing supersaturation increased $D_{50}$ by 43.0 and 25.0 µm, increasing seed loading raised it by 38.0 and 32.0 µm, and increasing power input reduced it by 18.0 and 13.0 µm, respectively. Figure 3B shows that longer residence time narrowed PSD span by 0.300 in both systems. Seed loading and power input also narrowed the distribution, whereas higher supersaturation broadened it, most strongly for carbamazepine (+0.350; p < 0.001).

**FIGURE 3:**
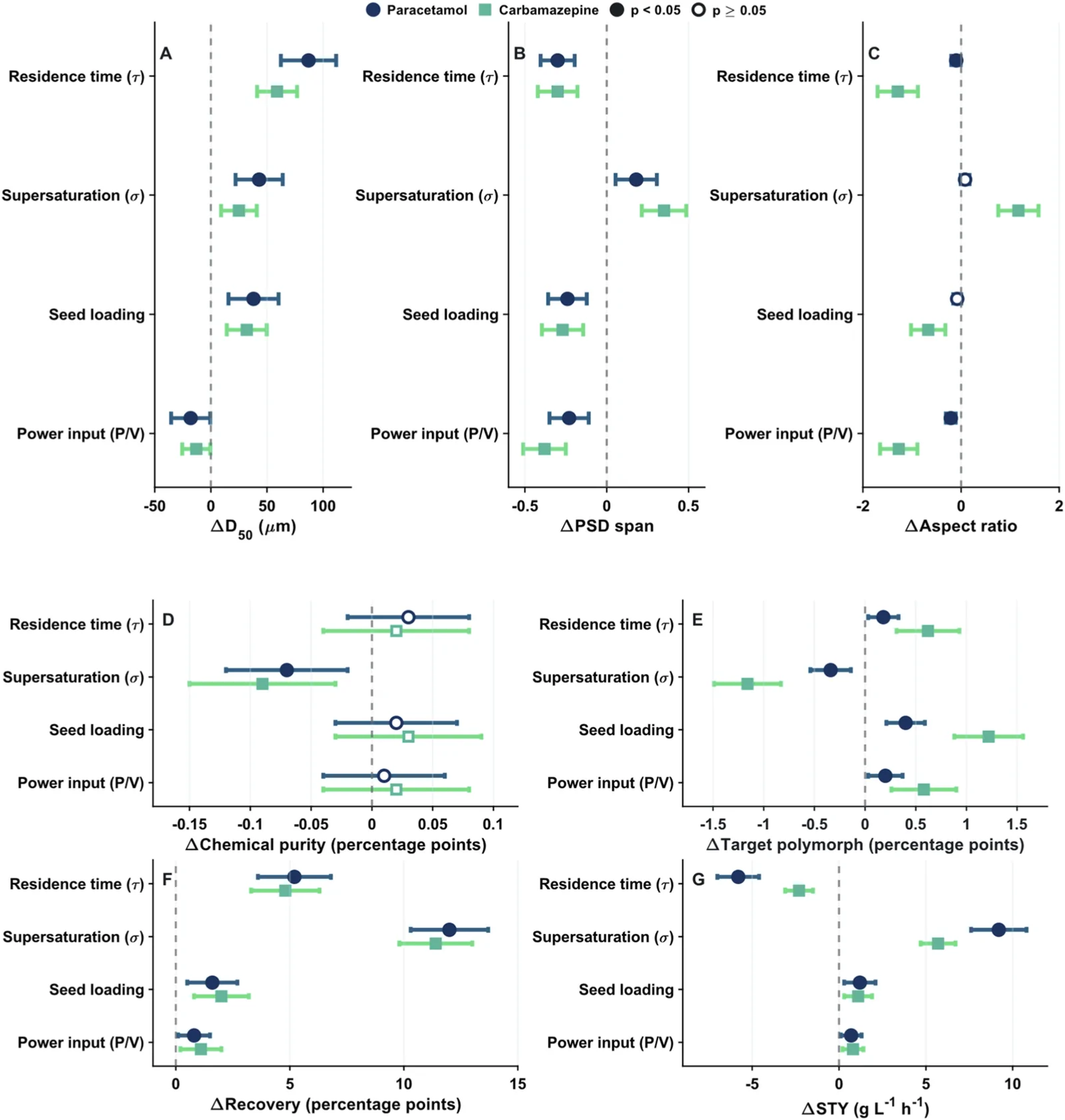
Effects of critical process parameters on particle quality and process performance. **(A)** Median particle size. **(B)** PSD span. **(C)** Aspect ratio. **(D)** Chemical purity. **(E)** Target polymorph. **(F)** Recovery. **(G)** Space-time yield. Points and error bars represent estimated changes and 95% CIs, respectively; filled symbols indicate p < 0.05 and open symbols indicate p ≥ 0.05. The dashed line denotes no change.

Chemical purity remained largely resistant to changes in the evaluated parameters. As shown in Figure 3D, residence time, seed loading, and power input produced no significant purity changes in either system. Increased supersaturation caused small but significant reductions of 0.07 percentage points for paracetamol and 0.09 percentage points for carbamazepine. In contrast, the target-polymorph fraction was more responsive, as demonstrated in Figure 3E. Seed loading produced the largest improvement, increasing the target polymorph by 0.40 percentage points for paracetamol and 1.22 percentage points for carbamazepine. Residence time and power input also increased the target-polymorph fraction, whereas supersaturation reduced it by 0.34 and 1.16 percentage points in paracetamol and carbamazepine, respectively.

Figure 3F shows that all four parameter increases significantly improved recovery. Supersaturation exerted the strongest effect, increasing recovery by 12.0 percentage points for paracetamol and 11.4 percentage points for carbamazepine. Longer residence time produced the next-largest gains of 5.2 and 4.8 percentage points, whereas seed loading and power input yielded smaller increases. The effects on space-time yield followed a different pattern. As shown in Figure 3G, increasing residence time reduced STY by 5.8 g L^‒1^ h^‒1^ for paracetamol and 2.3 g L^‒1^ h^‒1^ for carbamazepine, both with p < 0.001. Conversely, higher supersaturation generated the largest productivity gains, increasing STY by 9.2 and 5.7 g L^‒1^ h^‒1^, respectively. Seed loading and power input produced additional but smaller improvements. Figure 3 identifies a clear trade-off: longer residence time promoted particle growth, distribution narrowing, polymorphic control, and recovery at the expense of productivity, whereas higher supersaturation increased recovery and STY but broadened the PSD and reduced purity and target-polymorph content.

### 3.3 Response-surface optimization and experimental confirmation

All optimized conditions in Table 2 were confirmed in the 150-mL single-stage MSMPR. For paracetamol, the optimum produced $D_{50}$ = 248.6 ± 6.8 µm, target polymorph = 99.5 ± 0.2%, recovery = 19.4 ± 0.7%, STY = 16.46 ± 0.59 g L^‒1^ h^‒1^, and desirability = 0.899. Its robustness condition produced $D_{50}$ = 231.8 ± 7.4 µm, target polymorph = 99.1 ± 0.3%, recovery = 20.9 ± 0.8%, STY = 20.27 ± 0.78 g L^‒1^ h^‒1^, and desirability = 0.819. For carbamazepine, the optimum produced $D_{50}$ = 168.2 ± 5.9 µm, target polymorph = 99.1 ± 0.2%, recovery = 18.8 ± 0.7%, STY = 5.63 ± 0.21 g L^‒1^ h^‒1^, and desirability = 0.883. Its robustness condition produced $D_{50}$ = 157.7 ± 6.4 µm, target polymorph = 98.3 ± 0.3%, recovery = 21.1 ± 0.8%, STY = 7.19 ± 0.27 g L^‒1^ h^‒1^, and desirability = 0.796. Prediction errors were 1.5%–5.5% for $D_{50}$ , ≤0.9 percentage points for polymorph content, ≤0.9 percentage points for recovery, and 0.09–0.98 g L^‒1^ h^‒1^ for STY .

**TABLE 2:** Optimized conditions and experimental confirmation.

System–condition	Operating conditions*	$D_{50}$ (µm), P/O	Target polymorph (%), P/O	Recovery (%), P/O	STY (g L^‒1^ h^‒1^), P/O	Desirability, P/O
Paracetamol–optimum	33.6; 0.261; 1.08; 0.61; —	252.4/248.6 ± 6.8	99.6/99.5 ± 0.2	19.7/19.4 ± 0.7	16.72/16.46 ± 0.59	0.914/0.899
Paracetamol–robustness	29.4; 0.292; 1.15; 0.66; —	245.2/231.8 ± 7.4	99.6/99.1 ± 0.3	20.6/20.9 ± 0.8	19.29/20.27 ± 0.78	0.842/0.819
Carbamazepine–optimum	36.8; 0.253; 1.18; 0.64; 17.1	171.6/168.2 ± 5.9	99.3/99.1 ± 0.2	19.1/18.8 ± 0.7	5.72/5.63 ± 0.21	0.901/0.883
Carbamazepine–robustness	31.2; 0.291; 1.25; 0.70; 19.2	164.6/157.7 ± 6.4	99.2/98.3 ± 0.3	20.2/21.1 ± 0.8	7.49/7.19 ± 0.27	0.821/0.796

*Operating conditions are reported as τ (min), σ , seed (%), P/V (W L^‒1^), and water (% v/v), respectively. P/O, predicted/observed; observed results are mean ± SD.

The complete design matrix is reported in Supplementary Table S14, fitted coded equations in Supplementary Equations S1–S14, detailed ANOVA in Supplementary Table S15, and residual diagnostics in Supplementary Table S16 and Supplementary Figure S1. Across the 14 response models, $R^{2}$ ranged from 0.874 to 0.967, adjusted $R^{2}$ from 0.748 to 0.934, and predicted $R^{2}$ from 0.603 to 0.879. All overall models were significant (p ≤ 0.004), all lack-of-fit tests were nonsignificant (p = 0.184–0.412), and maximum VIF was 2.18.

Studentized residuals ranged from −2.21 to 2.34, Cook’s D remained ≤0.42, and neither normality nor homoscedasticity was rejected. The strongest interactions were residence time × supersaturation for recovery and STY , supersaturation × power input for $D_{50}$ and carbamazepine aspect ratio, and supersaturation × seed loading for target-polymorph content. Figure 4 visualizes these interactions and shows the feasible region defined by the QTPP constraints. Figure 4 was regenerated directly from Supplementary Equations S1–S14; the axes and responses are displayed in natural units, the fixed factor values are identified in each panel, and no response normalization or transformation was applied.

**FIGURE 4:**
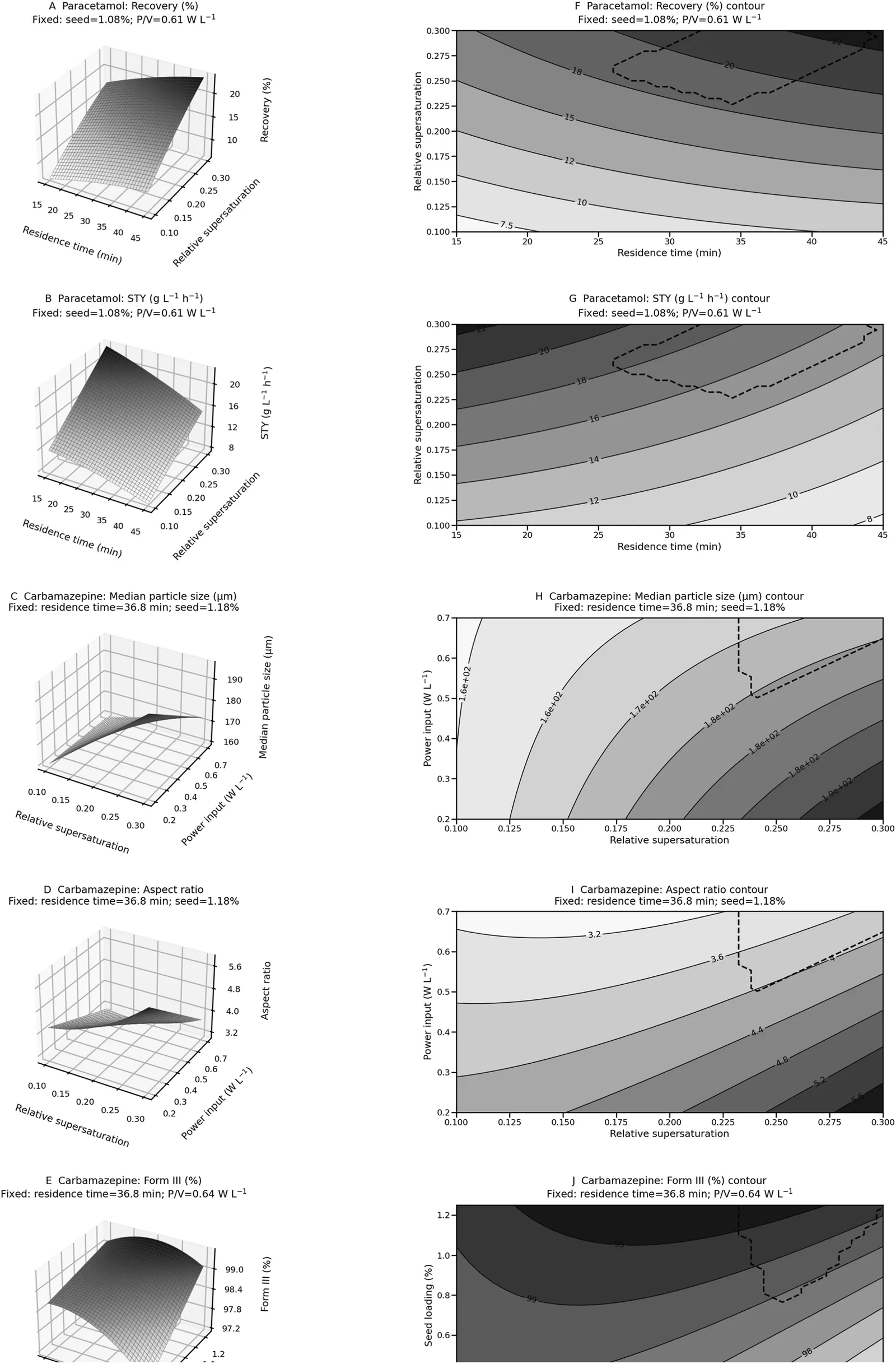
Response surfaces generated directly from Supplementary Equations S1–S14. Response surfaces **(A–E)** and corresponding contour plots **(F–J)** showing the effects of residence time, relative supersaturation, and power input on recovery, space-time yield (STY), median particle size, aspect ratio, and Form III content of carbamazepine under fixed operating conditions.

### 3.4 Population-balance modeling

#### 3.4.1 Estimated kinetic parameters

The estimated kinetic parameters revealed distinct crystallization behaviors for the two compounds. As shown in Table 3, paracetamol exhibited a higher growth-rate constant than carbamazepine, with ( $k_{g}$ ) values of 28.6 and 15.1 µm min^‒1^, respectively. Paracetamol also had an approximately 2.9-fold higher nucleation constant, with ( $k_{b}$ ) estimated at ( $3.42\times 10^{5}$ ) L^‒1^ min^‒1^ (g L^‒1^) ^−^ʲ compared with ( $1.18\times 10^{5}$ ) for carbamazepine. These estimates indicate faster intrinsic crystal growth and a greater nucleation-rate scale for paracetamol within the investigated operating domain. Carbamazepine showed higher growth, supersaturation, and suspension-density exponents. Table 3 reports growth exponents of 1.34 and 1.52, supersaturation exponents of 2.48 and 3.12, and suspension-density exponents of 1.12 and 1.31 for paracetamol and carbamazepine, respectively. Thus, although carbamazepine had lower kinetic constants, its growth and nucleation rates were more strongly dependent on the associated driving-force terms. All freely estimated parameters had relative standard errors below 17%, ranging from 5.6% to 16.8%, indicating acceptable estimation precision. The nucleus size was fixed at 2.0 µm for both models.

**TABLE 3:** Population-balance kinetic parameters estimated for paracetamol and carbamazepine.

Parameter	Paracetamol estimate (95% CI; RSE)	Carbamazepine estimate (95% CI; RSE)	Unit
Growth-rate constant, $k_{g}$	28.6 (24.1–33.8; RSE 8.5%)	15.1 (12.6–18.0; RSE 9.1%)	µm min^‒1^
Growth exponent, g	1.34 (1.20–1.49; RSE 5.6%)	1.52 (1.35–1.71; RSE 6.0%)	Dimensionless
Nucleation constant, $k_{b}$	3.42 × 10^5^ (2.48 × 10^5^–4.61 × 10^5^; RSE 14.2%)	1.18 × 10^5^ (0.79 × 10^5^–1.71 × 10^5^; RSE 16.8%)	L^‒1^ min^‒1^ (g L^‒1^) ^-^ʲ
Supersaturation exponent, b	2.48 (2.18–2.81; RSE 6.9%)	3.12 (2.72–3.58; RSE 7.4%)	Dimensionless
Suspension-density exponent, j	1.12 (0.94–1.31; RSE 8.4%)	1.31 (1.07–1.58; RSE 9.7%)	Dimensionless
Nucleus size, $L_{0}$	2.0 (fixed)	2.0 (fixed)	µm

#### 3.4.2 Calibration, internal validation, and external validation

The population-balance models reproduced particle-size, dissolved-concentration, and crystal-flow responses with consistently strong agreement across the three datasets. Figure 5 shows that calibration ( $R^{2}$ ) values ranged from 0.943 to 0.972, while NRMSE and MAPE remained within 1.2%–5.7% and 1.0%–4.9%, respectively. Predictive accuracy decreased modestly during internal and external validation, as expected for previously unseen conditions, but remained acceptable. Across both compounds and all outputs, external-validation ( $R^{2}$ ) values ranged from 0.894 to 0.943, with NRMSE values of 1.8%–8.8%. As shown in Figure 5A, the models accurately predicted $D_{50}$ across all datasets. For paracetamol, ( $R^{2}$ ) decreased moderately from 0.957 during calibration to 0.932 and 0.919 during internal and external validation, respectively, while NRMSE increased from 4.2% to 5.5% and 6.2%. Carbamazepine followed the same pattern, with corresponding ( $R^{2}$ ) values of 0.951, 0.925, and 0.911 and NRMSE values of 5.3%, 7.0%, and 7.9%. $D_{90}$ was the most challenging output to predict, particularly for carbamazepine. Figure 5B shows that external-validation NRMSE reached 7.0% for paracetamol and 8.8% for carbamazepine, with ( $R^{2}$ ) values of 0.902 and 0.894, respectively. Nevertheless, the models retained substantial explanatory performance for the upper particle-size distribution. The largest absolute external-validation biases were also observed for $D_{90}$ , at −6.4 µm for paracetamol and −4.5 µm for carbamazepine. Dissolved concentration was predicted most accurately. As demonstrated in Figure 5C, the paracetamol model achieved ( $R^{2}$ ) values of 0.972, 0.951, and 0.943 across calibration, internal validation, and external validation, respectively, with NRMSE remaining below 2%. The corresponding carbamazepine ( $R^{2}$ ) values were 0.968, 0.947, and 0.936, with a maximum NRMSE of 3.0%. Figure 5D further shows that crystal-flow predictions remained reliable, with external-validation ( $R^{2}$ ) values of 0.925 for paracetamol and 0.918 for carbamazepine and NRMSE values of 4.8% and 5.7%, respectively. Mean biases remained small relative to the magnitudes of the modeled outputs and changed direction between internal validation and the other datasets. Overall, Figure 5 demonstrates that the models retained good predictive performance beyond the calibration data, with dissolved concentration showing the greatest accuracy and $D_{90}$ showing the largest, although still limited, validation error.

**FIGURE 5:**
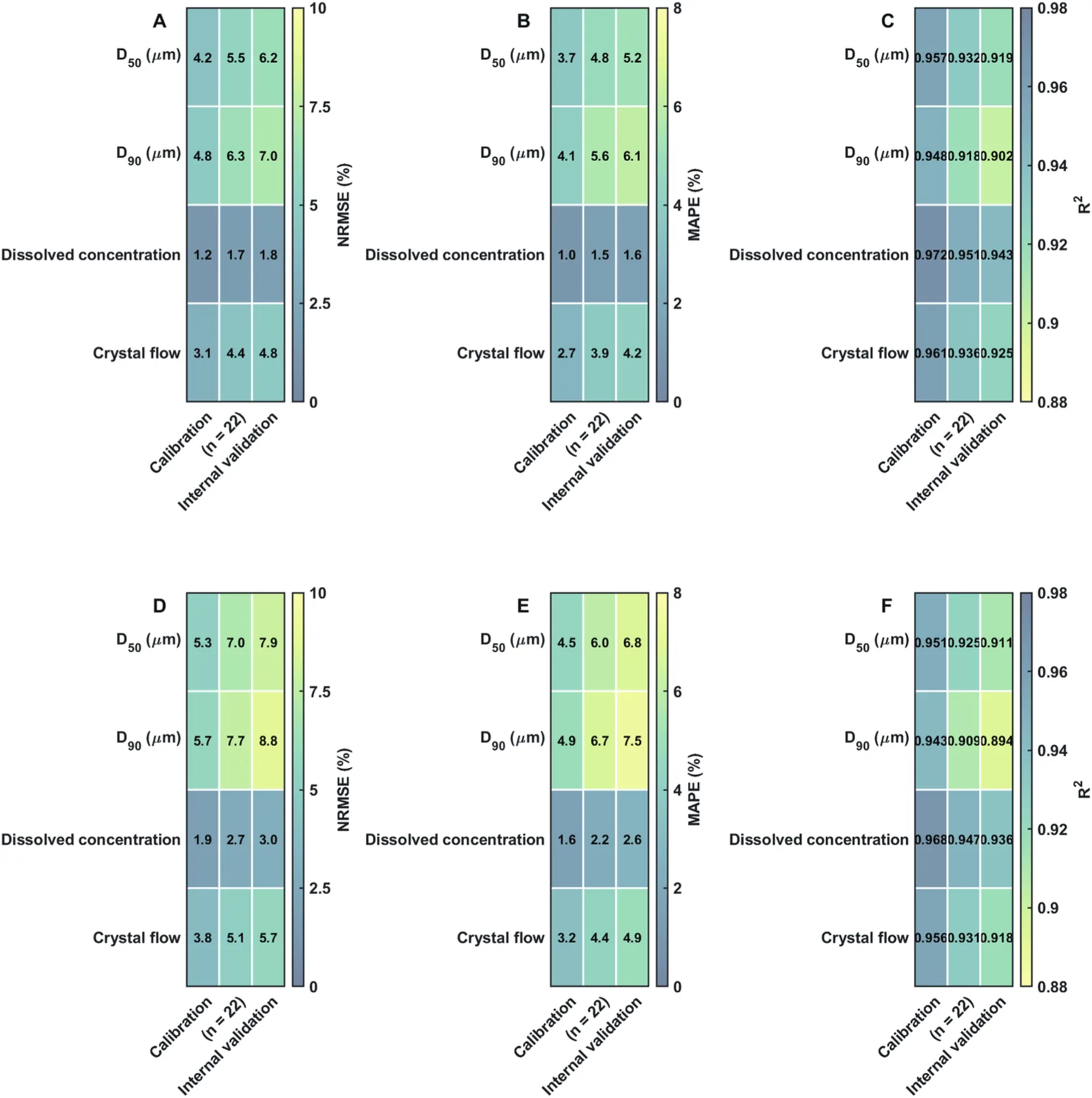
Calibration and validation performance of the population‐balance models. Panels **(A, D)** show normalized root mean square error (NRMSE), **(B, E)** show mean absolute percentage error (MAPE), and **(C, F)** show the coefficient of determination (R^2^) for $D_{50}$ , $D_{90}$ , dissolved concentration, and crystal flow.

### 3.5 Comparison of single-stage and two-stage MSMPR configurations

The two-stage MSMPR configuration yielded larger and more uniformly distributed particles in both systems. Figure 6A shows that, for paracetamol, $D_{10}$ , $D_{50}$ , and $D_{90}$ increased by 25.6%, 15.8%, and 4.9%, respectively, while the PSD span decreased by 14.6%; all differences remained significant after Holm adjustment. The aspect ratio decreased by 3.4%, although this change was not significant. Recovery and STY each increased by 3.6%, whereas chemical purity, Form I content, and mass-balance closure remained statistically unchanged.

**FIGURE 6:**
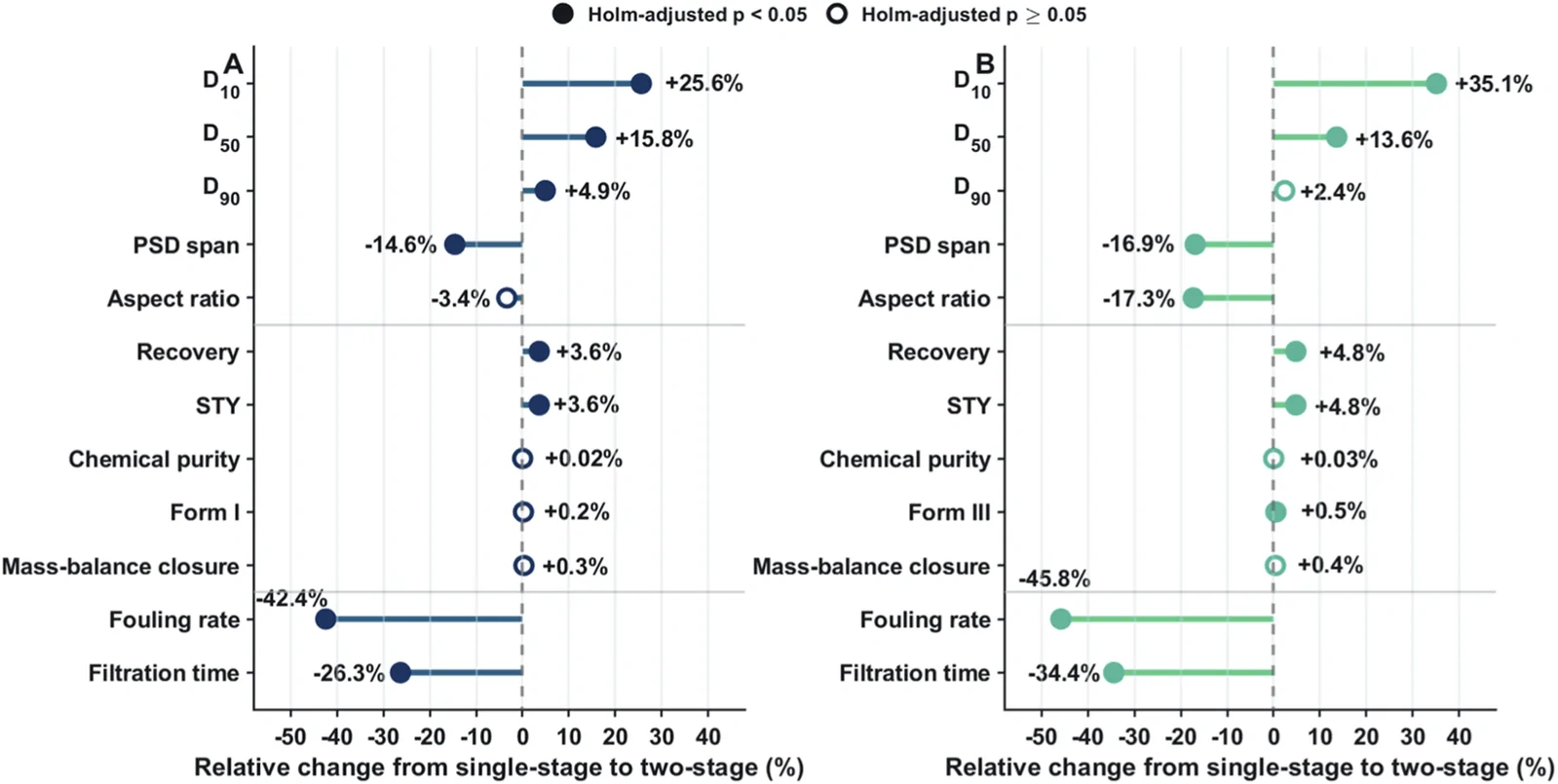
*Relative changes from single-stage to two-stage MSMPR operation.* **(A)** *Paracetamol.* **(B)** *Carbamazepine. Filled and open symbols denote Holm-adjusted p < 0.05 and p ≥ 0.05, respectively; the dashed line indicates no change.*

As shown in Figure 6B, two-stage carbamazepine crystallization increased $D_{10}$ and $D_{50}$ by 35.1% and 13.6%, respectively. The 2.4% increase in $D_{90}$ was not significant. The PSD span and aspect ratio decreased significantly by 16.9% and 17.3%, indicating a narrower particle-size distribution and less elongated morphology. Recovery and STY each increased by 4.8%, while Form III content increased from 99.1% ± 0.2% to 99.6% ± 0.2%. Chemical purity and mass-balance closure did not differ significantly between the configurations. The greatest operational improvements involved fouling and filtration. Figure 6A demonstrates reductions of 42.4% in fouling rate and 26.3% in filtration time for paracetamol. Figure 6B shows corresponding reductions of 45.8% and 34.4% for carbamazepine. Overall, the two-stage configuration produced larger, more uniform particles and substantially improved downstream processing while preserving chemical purity and mass-balance closure.

### 3.6 Agreement between online process-analytical and offline reference methods

Table 4 demonstrates strong agreement between the online PAT measurements and their corresponding offline reference methods, with calibration $R^{2}$ values of 0.903–0.996, CCCs of 0.944–0.995, and validation slopes of 0.921–0.993. Spectroscopic concentration models performed best: paracetamol ATR-FTIR/PLS achieved ( $R^{2}=0.996$ ), RMSEP of 0.71 g L^‒1^, CCC of 0.995, and a bias of −0.08 g L^‒1^, whereas carbamazepine Raman/PLS achieved ( $R^{2}=0.995$ ), RMSEP of 0.36 g L^‒1^, CCC of 0.994, and a bias of +0.05 g L^‒1^. Raman monitoring of carbamazepine Form III also showed high accuracy, with ( $R^{2}=0.982$ ), RMSEP of 0.46 percentage points, CCC of 0.986, and limits of agreement within approximately ±1 percentage point. FBRM-derived ( $D_{50}$ ) estimates showed slightly lower but still strong agreement, with CCCs of 0.957 and 0.951 and RMSEP values of 12.6 and 9.4 µm for paracetamol and carbamazepine, respectively. Video microscopy reproduced aspect ratio with CCCs of 0.944 and 0.969 and low biases of +0.02 and +0.04. Overall, the methods provided low-bias, strongly concordant estimates of concentration, solid form, particle size, and morphology, supporting their use for real-time monitoring and control.

**TABLE 4:** Agreement between online process-analytical measurements and offline reference methods.

System	Online method and response	Offline References	Calibration/validation n	Analytical range	Calibration $R^{2}$	RMSEC	RMSEP	Validation bias	Slope	Intercept	95% limits of agreement	CCC
Paracetamol	ATR-FTIR/PLS concentration	HPLC	48/18	10–48 g L^‒1^	0.996	0.46 g L^‒1^	0.71 g L^‒1^	−0.08 g L^‒1^	0.993	0.21	−1.43 to 1.27 g L^‒1^	0.995
	FBRM-derived $D_{50}$	Laser diffraction	42/18	80–330 µm	0.927	10.4 µm	12.6 µm	−2.1 µm	0.940	9.8	−25.8 to 21.6 µm	0.957
	Video-microscopy aspect ratio	Offline image analysis	36/15	1.30–1.85	0.903	0.09	0.11	+0.02	0.921	0.14	−0.20 to 0.24	0.944
Carbamazepine	Raman/PLS concentration	HPLC	54/20	2–22 g L^‒1^	0.995	0.27 g L^‒1^	0.36 g L^‒1^	+0.05 g L^‒1^	0.989	0.16	−0.66–0.76 g L^‒1^	0.994
	Raman form III content	Quantitative XRPD	45/18	94%–100%	0.982	0.31 pp	0.46 pp	−0.07 pp	0.972	2.70	−0.97 to 0.83 pp	0.986
	FBRM-derived $D_{50}$	Laser diffraction	44/18	65–210 µm	0.918	7.8 µm	9.4 µm	−1.6 µm	0.932	8.9	−20.0 to 16.8 µm	0.951
	Video-microscopy aspect ratio	Offline image analysis	38/16	2.8–6.2	0.941	0.18	0.23	+0.04	0.956	0.16	−0.41 to 0.49	0.969

### 3.7 Fouling, filtration, and extended operational robustness

As shown in Table 5, the two-stage MSMPR configuration improved fouling control, filtration performance, and extended operational robustness for both compounds. For paracetamol, fouling decreased by 42.4%, from 0.66 ± 0.08 to 0.38 ± 0.06 g m^‒2^ h^‒1^, while product loss declined from 0.96% to 0.64%. Filtration time and cake resistance were reduced by 26.3% and 31.9%, respectively. Carbamazepine remained the more filtration-limited system but showed larger relative gains: fouling decreased by 45.8%, from 0.83 ± 0.09 to 0.45 ± 0.07 g m^‒2^ h^‒1^, product loss fell from 3.54% to 2.19%, filtration time decreased by 34.4%, and cake resistance by 37.4%. No operational interruptions occurred. Two-stage operation also reduced variability in flow, $D_{50}$ , and concentration from 1.1/2.8/1.9% to 0.8/2.1/1.5% for paracetamol and from 1.6/3.5/2.6% to 1.0/2.4/1.8% for carbamazepine. Minimum target-polymorph content increased from 99.3% to 99.5% and from 98.8% to 99.3%, respectively. Overall, staging reduced deposition, product loss, filtration resistance, and process variability while maintaining high polymorphic stability.

**TABLE 5:** Fouling, filtration, and extended operational robustness at the optimized conditions.

System	Configuration	Fouling rate (g m^‒2^ h^‒1^)	Product loss (%)	Filtration time (s/100 mL)	Cake resistance (×10^10^ m kg^‒1^)	CV: flow/ $D_{50}$ /concentration (%)	Minimum target polymorph (%)	Interruptions
Paracetamol	Single-stage	0.66 ± 0.08	0.96	42.6 ± 3.2	1.82 ± 0.16	1.1/2.8/1.9	99.3	0
	Two-stage	0.38 ± 0.06	0.64	31.4 ± 2.7	1.24 ± 0.12	0.8/2.1/1.5	99.5	
Carbamazepine	Single-stage	0.83 ± 0.09	3.54	96.8 ± 6.4	4.76 ± 0.35	1.6/3.5/2.6	98.8	
	Two-stage	0.45 ± 0.07	2.19	63.5 ± 5.1	2.98 ± 0.27	1.0/2.4/1.8	99.3	

## 4 Discussion

The present study demonstrates that continuous pharmaceutical crystallization in MSMPR systems is controlled by interacting thermodynamic, kinetic, hydrodynamic, and solid-state factors. Figure 3 quantifies the CPP effects, Table 2 confirms the optimized single-stage conditions, Figure 5 validates the population-balance predictions, and Figure 6 and Table 5 show the configuration-dependent operational gains. The principal advance over earlier MSMPR studies is the matched comparison at identical total active volume and overall residence time for two APIs, integrated with external model validation, PAT concordance (Table 4), and downstream performance. Accordingly, the work provides a reproducible compound-specific workflow, not a universal MSMPR framework (Ma et al., 2020; Orehek et al., 2020; Barhate et al., 2024; Aprile et al., 2025; Eren et al., 2023).

The solubility and metastable-zone data in Figure 2 explain the different process responses. Paracetamol’s strong temperature dependence supports cooling crystallization, whereas its wider metastable zone at faster cooling permits greater supersaturation accumulation and therefore raises fines risk under local thermal gradients (Ma et al., 2020; Sarkar et al., 2025). Carbamazepine was more sensitive to solvent composition: increasing water fraction sharply reduced solubility, so poor micromixing can generate local water-rich zones, excessive nucleation, elongated crystals, and polymorphic heterogeneity (Orehek et al., 2020; Eren et al., 2023; Sarkar et al., 2025). The process-parameter analysis in Figures 3A,B,E,F, and G revealed a consistent quality–productivity trade-off. Longer residence time increased $D_{50}$ , narrowed the PSD, improved target-polymorph content, and increased recovery, but reduced STY . Mechanistically, longer exposure increased solute deposition on established crystal surfaces and reduced the relative contribution of newly formed fines. The negative STY effect follows directly from lower throughput at fixed crystallizer volume. These coupled effects are consistent with growth-dominated operation and previous MSMPR studies (Ma et al., 2020; Pascual et al., 2022; Sarkar et al., 2025; Stoffan et al., 2024).

Supersaturation generated the opposite response (Figures 3B–G): it produced the largest recovery and STY gains but broadened the PSD, slightly reduced chemical purity, decreased target-polymorph content, and strongly increased carbamazepine aspect ratio. The higher driving force raises primary and secondary nucleation rates more steeply than growth, increasing fines and broadening residence-dependent crystal histories. Carbamazepine’s stronger morphology response is consistent with anisotropic face growth under local water-rich, high-supersaturation conditions. The small purity losses likely reflect mother-liquor inclusion, fines retention, surface adsorption, or reduced impurity rejection during rapid growth (Ma et al., 2020; Orehek et al., 2020; Eren et al., 2023; Liu et al., 2024).

Continuous seeding moderated these trade-offs by providing reproducible growth surfaces and structural templates. Higher seed loading increased $D_{50}$ , narrowed the PSD, improved recovery and STY , and enhanced target-polymorph content, particularly for carbamazepine. This result is consistent with suppression of stochastic primary nucleation and promotion of surface-mediated secondary nucleation of the intended form. However, seed loading must remain balanced: insufficient seeding permits uncontrolled nucleation, whereas excessive seed numbers may distribute the available solute across too much surface area and limit final crystal growth (Orehek et al., 2020; Pascual et al., 2022; Eren et al., 2023; Stoffan et al., 2024).

Power input exerted mixed effects (Figure 3). Increasing P/V reduced $D_{50}$ but narrowed the PSD, decreased aspect ratio, improved polymorphic purity, and modestly increased recovery and STY . The size reduction may reflect attrition, breakage, or enhanced secondary nucleation; the narrower PSD and lower aspect ratio are consistent with better suspension and micromixing. This hydrodynamic effect was strongest for carbamazepine, although excessive mechanical stress can still increase fines (Liu et al., 2024). Response-surface optimization integrated these competing effects. Table 2 shows close prediction–confirmation agreement, while Supplementary Tables S8, S9, and Supplementary Tables S8, S9–S16 establish model significance, nonsignificant lack of fit, acceptable residual behavior, and low collinearity. Figure 4 shows that high recovery and STY occurred along the high-supersaturation boundary, whereas the QTPP-constrained optimum remained at intermediate supersaturation with relatively high seeding and power input. The robustness conditions deliberately shifted toward throughput and therefore reduced particle size, polymorphic purity, and overall desirability (Pascual et al., 2022; Szilagyi et al., 2020; Stoffan et al., 2024).

The population-balance parameters in Table 3 explain the compound-specific behavior. Paracetamol had larger fitted growth and nucleation constants, whereas carbamazepine had higher supersaturation and suspension-density exponents. Thus, paracetamol exhibited a higher kinetic rate scale within the studied domain, while carbamazepine was more sensitive to driving force and solids loading. Direct constant-to-constant comparison remains cautious because solvents and crystallization modes differed. Acceptable parameter precision, bootstrap uncertainty, and the external predictions in Figure 5 provide the stronger evidence for mechanistic credibility (Barhate et al., 2024). External validation retained $R^{2}$ = 0.894–0.943 and NRMSE <9%. Dissolved concentration was predicted most accurately because it is constrained by the liquid-phase mass balance; $D_{90}$ had the largest error because the upper PSD tail is sensitive to rare large particles, agglomeration, growth-rate dispersion, and nonideal solid RTD. The remaining underprediction motivates size-dependent growth, agglomeration, breakage, and multidimensional PBMs for anisotropic carbamazepine (Barhate et al., 2024; Liu et al., 2024).

The two-stage MSMPR configuration produced the most practically important improvements (Figure 6; Table 5). For both compounds, the increase in $D_{10}$ exceeded the increase in $D_{50}$ , showing that staging primarily suppressed the fines fraction. Splitting cooling or antisolvent addition reduced the maximum supersaturation imposed in either vessel and allowed particles generated or seeded in stage 1 to grow under moderated driving force before final desupersaturation. This redistributed residence time across two supersaturation environments and shifted the population balance from nucleation-dominated toward growth-dominated operation (Orehek et al., 2020; Amari et al., 2022; Eren et al., 2023; Stoffan et al., 2024). The carbamazepine result was particularly informative: at matched volume and residence time, staging reduced aspect ratio by 17.3%, narrowed the PSD, increased Form III content, and improved recovery and STY . Because capacity was controlled, these gains arise from the altered solvent-composition and supersaturation histories rather than added equipment volume. Dividing antisolvent addition also limits local water-rich zones and high-driving-force needle growth (Amari et al., 2022; Stoffan et al., 2024).

The recovery and STY gains from staging were moderate but meaningful because they coincided with improved particle quality. Figure 6 shows increases of 3.6% for paracetamol and 4.8% for carbamazepine. Single-stage isolated recoveries of 19.4% and 18.8% agreed with equilibrium-depletion predictions of 20.8% and 19.9%, respectively, within 1.4 and 1.1 percentage points. The five-sample steady-state criteria were satisfied before collection, concentration slopes were nonsignificant, and mass-balance closure was 99.2%–99.7%; the low recoveries therefore reflected residual equilibrium solubility rather than failure to reach steady state. Mother-liquor recycle, additional stages, or solvent removal remain logical routes to higher recovery (Orehek et al., 2020; Barhate et al., 2024; Eren et al., 2023). The PAT results in Table 4 confirm analytical observability. ATR-FTIR and Raman concentration models had near-unity slopes, minimal bias, and concordance correlation coefficients above 0.99. Raman also quantified carbamazepine Form III. FBRM and video microscopy showed slightly lower concordance but remained useful trend and morphology sensors; FBRM was not treated as a laser-diffraction substitute (Domokos et al., 2021; Pal et al., 2024; Zhang et al., 2024; Sarkar et al., 2025; Gao et al., 2021). Finally, Figure 6 and Table 5 link the larger, narrower particles and lower carbamazepine aspect ratio to reduced deposition, product loss, filtration time, and cake resistance. Lower local supersaturation near walls, probes, and inlets reduces heterogeneous nucleation, while fewer fines create more permeable filter cakes (Acevedo et al., 2019; Aprile et al., 2025; Liu et al., 2024).

Extended-operation data in Table 5 further supported cascade robustness. No interruption occurred, mass-balance closure remained acceptable, and the target polymorph was maintained in every configuration. In addition, two-stage operation reduced the coefficients of variation for flow and concentration. The second stage therefore attenuated short disturbances and provided additional relaxation time for the dissolved concentration and particle population. The higher minimum polymorph content, particularly for carbamazepine, indicates reduced transient solid-form risk (Gao et al., 2021; Nandan and Parambil, 2023).

Several limitations remain. The total active volume was 150 mL, so scale-up may alter mixing, energy dissipation, heat transfer, suspension, and withdrawal. Only two APIs and solvent systems were studied; compounds with stronger agglomeration, slower kinetics, solvates, hydrates, or more competitive polymorphism may behave differently. The one-dimensional PBM simplified carbamazepine anisotropy, and extended runs did not establish campaign-scale fouling over days or weeks. Economic and environmental effects of the additional vessel, PAT instruments, and solvent handling were not assessed. Future work should compare the present 60:40 duty split with sequential stage set-points that re-establish the desired supersaturation in each vessel, repeat the full DoE in both single-stage and two-stage hardware, and examine hydrodynamically informed scale-up, longer campaigns, recycle, disturbances, multidimensional PBMs, closed-loop control, and technoeconomic performance.

## 5 Conclusion

Continuous MSMPR crystallization can be developed into a predictable and robust pharmaceutical particle-engineering operation by integrating thermodynamic characterization, multivariable experimentation, mechanistic modeling, PAT, and downstream processability. Residence time favored crystal growth, PSD narrowing, recovery, and solid-form control but reduced STY , whereas supersaturation enhanced productivity at the expense of morphology and polymorphic selectivity. Seeding and power input moderated these competing effects. Most importantly, two-stage operation produced larger and more uniform particles, improved carbamazepine morphology and Form III control, reduced fouling by more than 40%, shortened filtration time, lowered cake resistance, and decreased process variability without compromising purity or mass-balance closure. These findings establish staged MSMPR crystallization as a promising platform for simultaneous control of product quality, productivity, and operational robustness.

## Data Availability

The original contributions presented in the study are included in the article/Supplementary Material, further inquiries can be directed to the corresponding author.
