# Supplementary material for: Continuous crystallization of pharmaceutical compounds in mixed suspension mixed product removal systems
Source: Front Chem. 2026 Oct 16;14:1947756. doi: 10.3389/fchem.2026.1947756 (PMC13721075; doi:10.3389/fchem.2026.1947756)
Supplement: Supplementary file 1 [file DataSheet1.docx]

**Supplementary**

***Supplementary Table S1. Complete physicochemical, equilibrium-solubility, and metastable-zone data for the paracetamol system (numerical source data for Figure 2)***

***A. Initial solid-state and seed characterization***

| **Attribute** | **As-received paracetamol** | **Qualified Form I seeds** |
| --- | --- | --- |
| HPLC assay (%) | 99.72 ± 0.05 | 99.69 ± 0.06 |
| Water content (%) | 0.18 ± 0.02 | 0.15 ± 0.02 |
| Target phase by quantitative XRPD (%) | 99.5 ± 0.3 | 99.8 ± 0.2 |
| DSC melting onset (°C) | 169.3 ± 0.4 | 169.4 ± 0.3 |
| TGA mass loss before melting (%) | 0.21 ± 0.03 | 0.17 ± 0.02 |
| $D₁₀$ (µm) | 42.6 ± 2.1 | 78.6 ± 2.8 |
| $D₅₀$ (µm) | 87.3 ± 3.4 | 99.4 ± 3.1 |
| $D₉₀$ (µm) | 156.8 ± 5.7 | 122.7 ± 4.5 |
| PSD span | 1.308 | 0.444 |
| Mean aspect ratio | 1.71 ± 0.31 | 1.62 ± 0.28 |

***Supplementary Table S1 (continued). Complete physicochemical, equilibrium-solubility, and metastable-zone data for the paracetamol system (numerical source data for Figure 2)***

***B. Equilibrium solubility of paracetamol in water***

| **Temperature (°C)** | **Solubility (g L⁻¹)** | **Solubility (mol L⁻¹)** | **RSD (%)** |
| --- | --- | --- | --- |
| 10 | 8.82 ± 0.14 | 0.0583 | 1.59 |
| 15 | 10.47 ± 0.16 | 0.0693 | 1.53 |
| 20 | 13.08 ± 0.19 | 0.0865 | 1.45 |
| 25 | 16.41 ± 0.23 | 0.1086 | 1.40 |
| 30 | 20.62 ± 0.31 | 0.1364 | 1.50 |
| 40 | 34.08 ± 0.47 | 0.2255 | 1.38 |
| 50 | 52.80 ± 0.69 | 0.3493 | 1.31 |

***Supplementary Table S1 (continued). Complete physicochemical, equilibrium-solubility, and metastable-zone data for the paracetamol system (numerical source data for Figure 2)***

***C. Metastable-zone characteristics***

| **Cooling rate (°C min⁻¹)** | **Saturation temperature (°C)** | **Nucleation temperature (°C)** | **MZW,** $\Delta T$ **(°C)** | **Cooling interval (min)** | $c\_eq$ **at nucleation (g L⁻¹)** | $S\_nuc$ | $\sigma\_nuc$ |
| --- | --- | --- | --- | --- | --- | --- | --- |
| 0.20 | 48.1 ± 0.2 | 38.8 ± 0.4 | 9.3 ± 0.5 | 46.5 | 31.52 | 1.508 | 0.508 |
| 0.50 |  | 36.4 ± 0.5 | 11.7 ± 0.6 | 23.4 | 27.52 | 1.727 | 0.727 |
| 1.00 |  | 33.0 ± 0.6 | 15.1 ± 0.7 | 15.1 | 23.20 | 2.048 | 1.048 |

***Note. Values are mean ± SD (n=3). PSD span =*** $(D₉₀-D₁₀)/D₅₀$***. The tabulated values are the numerical source data used for the analyses and figures reported in the manuscript.***

***Supplementary Table S2. Complete physicochemical, equilibrium-solubility, and metastable-zone data for the carbamazepine system (numerical source data for Figure 2)***

***A. Initial solid-state and seed characterization***

| Attribute | As-received carbamazepine | Qualified Form III seeds |
| --- | --- | --- |
| HPLC assay (%) | 99.58 ± 0.07 | 99.61 ± 0.06 |
| Water content (%) | 0.11 ± 0.02 | 0.09 ± 0.01 |
| Residual ethanol (%) | 0.032 ± 0.006 | 0.041 ± 0.007 |
| Form III content by quantitative XRPD (%) | 98.9 ± 0.4 | 99.7 ± 0.2 |
| DSC melting onset (°C) | 190.8 ± 0.5 | 191.0 ± 0.4 |
| TGA mass loss before melting (%) | 0.14 ± 0.03 | 0.13 ± 0.02 |
| $D₁₀$ (µm) | 34.7 ± 2.0 | 77.9 ± 2.6 |
| $D₅₀$ (µm) | 76.5 ± 3.2 | 100.7 ± 3.4 |
| $D₉₀$ (µm) | 148.9 ± 5.9 | 124.8 ± 4.2 |
| PSD span | 1.493 | 0.466 |
| Mean aspect ratio | 4.82 ± 1.09 | 3.91 ± 0.72 |

***Supplementary Table S2 (continued). Complete physicochemical, equilibrium-solubility, and metastable-zone data for the carbamazepine system (numerical source data for Figure 2)***

***B. Equilibrium solubility of carbamazepine in ethanol–water mixtures***

| **Water fraction (% v/v)** | **15 °C (g L⁻¹)** | **20 °C (g L⁻¹)** | **25 °C (g L⁻¹)** | **Solubility reduction at 15 °C relative to ethanol (%)** |
| --- | --- | --- | --- | --- |
| 0 | 21.00 ± 0.28 | 23.38 ± 0.31 | 25.80 ± 0.35 | 0.0 |
| 10 | 17.20 ± 0.24 | 19.15 ± 0.27 | 21.45 ± 0.30 | 18.1 |
| 20 | 13.42 ± 0.19 | 15.25 ± 0.22 | 17.35 ± 0.25 | 36.1 |
| 30 | 9.50 ± 0.15 | 11.05 ± 0.17 | 12.85 ± 0.20 | 54.8 |
| 40 | 6.20 ± 0.11 | 7.38 ± 0.13 | 8.72 ± 0.15 | 70.5 |
| 50 | 3.62 ± 0.07 | 4.45 ± 0.09 | 5.40 ± 0.11 | 82.8 |
| 60 | 1.82 ± 0.04 | 2.30 ± 0.05 | 2.88 ± 0.06 | 91.3 |

***Supplementary Table S2 (continued). Complete physicochemical, equilibrium-solubility, and metastable-zone data for the carbamazepine system (numerical source data for Figure 2)***

***C. Metastable-zone characteristics at 30% v/v water***

| **Cooling rate (°C min⁻¹)** | **Saturation temperature (°C)** | **Nucleation temperature (°C)** | **MZW,** $\Delta T$ **(°C)** | **Cooling interval (min)** | $c\_eq$ **at nucleation (g L⁻¹)** | $S\_nuc$ | $\sigma\_nuc$ |
| --- | --- | --- | --- | --- | --- | --- | --- |
| 0.20 | 35.2 ± 0.3 | 24.4 ± 0.4 | 10.8 ± 0.5 | 54.0 | 12.65 | 1.383 | 0.383 |
| 0.50 |  | 21.8 ± 0.5 | 13.4 ± 0.6 | 26.8 | 11.61 | 1.507 | 0.507 |
| 1.00 |  | 18.6 ± 0.6 | 16.6 ± 0.7 | 16.6 | 10.71 | 1.634 | 0.634 |

***Supplementary Table S3. Hydrodynamic qualification and residence-time-distribution parameters of the MSMPR systems***

| **Configuration** | **Total nominal** $\tau$ **(min)** | **Stage** $\tau$ **(min)** | $P/V$ **(W L⁻¹)** | $t₉₅$ **per stage (min)** | $t₉₅/\tau\_stage$ **(%)** | **Liquid mean RT (min)** | **Liquid RT deviation (%)** | **Solid mean RT (min)** | **Solid RT deviation (%)** | **Normalized RTD variance** | **Tanks-in-series number** | **Tracer recovery (%)** |
| --- | --- | --- | --- | --- | --- | --- | --- | --- | --- | --- | --- | --- |
| Single-stage | 15 | 15 | 0.20 | 1.28 ± 0.06 | 8.53 | 14.6 ± 0.4 | −2.67 | 15.2 ± 0.5 | +1.33 | 0.93 | 1.08 | 98.7 ± 0.6 |
|  | 30 | 30 | 0.45 | 1.72 ± 0.08 | 5.73 | 29.5 ± 0.7 | −1.67 | 30.4 ± 0.8 |  | 0.89 | 1.12 | 99.2 ± 0.4 |
|  | 45 | 45 | 0.70 | 2.14 ± 0.10 | 4.76 | 44.1 ± 1.0 | −2.00 | 45.7 ± 1.1 | +1.56 | 0.86 | 1.16 | 99.0 ± 0.5 |
| Two-stage | 15 | 7.5 + 7.5 | 0.20 | 0.69 ± 0.04 | 9.20 | 15.1 ± 0.4 | +0.67 | 15.5 ± 0.5 | +3.33 | 0.52 | 1.92 | 98.8 ± 0.5 |
|  | 30 | 15 + 15 | 0.45 | 0.91 ± 0.05 | 6.07 | 30.5 ± 0.7 | +1.67 | 31.1 ± 0.8 | +3.67 | 0.49 | 2.04 | 99.3 ± 0.4 |
|  | 45 | 22.5 + 22.5 | 0.70 | 1.18 ± 0.06 | 5.24 | 45.8 ± 1.0 | +1.78 | 46.6 ± 1.1 | +3.56 | 0.47 | 2.13 | 99.1 ± 0.4 |

***Supplementary Table S4. Representative steady-state performance of continuous paracetamol cooling crystallization***

| **Run** | $\tau$ **(min)** | $\sigma$ | **Seed loading (%)** | $P/V$ **(W L⁻¹)** | **Outlet temperature (°C)** | **Time to steady state (**$\tau$**)** | **Mother-liquor concentration (g L⁻¹)** | $D₅₀$ **(µm)** | **Recovery (%)** | **Crystal flow (mg min⁻¹)** | $STY$ **(g L⁻¹ h⁻¹)** | **Mass-balance closure (%)** | **HPLC purity (%)** | **Form I (%)** |
| --- | --- | --- | --- | --- | --- | --- | --- | --- | --- | --- | --- | --- | --- | --- |
| P-08 | 15 | 0.10 | 0.75 | 0.45 | 44.9 ± 0.1 | 5.6 | 43.67 ± 0.52 | 118.4 ± 5.6 | 8.1 ± 0.6 | 38.49 | 15.40 | 99.2 ± 1.4 | 99.76 ± 0.05 | 99.6 ± 0.2 |
| P-C | 30 | 0.20 |  |  | 43.0 ± 0.1 | 5.8 | 40.20 ± 0.44 | 203.9 ± 7.8 | 15.4 ± 0.8 | 36.59 | 14.64 | 100.1 ± 1.1 | 99.72 ± 0.06 | 99.5 ± 0.2 |
| P-21 | 45 | 0.30 |  |  | 41.3 ± 0.1 | 6.2 | 37.16 ± 0.41 | 318.6 ± 10.9 | 21.8 ± 1.0 | 34.53 | 13.81 | 98.8 ± 1.5 | 99.65 ± 0.07 | 99.1 ± 0.3 |

***Supplementary Table S5. Representative steady-state performance of carbamazepine antisolvent-assisted crystallization***

| **Run** | $\tau$ **(min)** | $\sigma$ | **Water fraction (% v/v)** | **Seed loading (%)** | $P/V$ **(W L⁻¹)** | **Time to steady state (**$\tau$**)** | **Mother-liquor concentration (g L⁻¹)** | $D₅₀$ **(µm)** | **Aspect ratio** | **Recovery (%)** | **Crystal flow (mg min⁻¹)** | $STY$ **(g L⁻¹ h⁻¹)** | **Mass-balance closure (%)** | **HPLC purity (%)** | **Form III (%)** |
| --- | --- | --- | --- | --- | --- | --- | --- | --- | --- | --- | --- | --- | --- | --- | --- |
| C-07 | 15 | 0.10 | 5.9 | 0.75 | 0.45 | 6.1 | 19.05 ± 0.29 | 83.6 ± 4.8 | 5.25 ± 0.51 | 7.7 ± 0.6 | 15.89 | 6.36 | 98.9 ± 1.6 | 99.70 ± 0.06 | 99.4 ± 0.2 |
| C-C | 30 | 0.20 | 13.8 |  |  | 6.4 | 16.09 ± 0.26 | 132.4 ± 6.7 | 4.58 ± 0.42 | 14.9 ± 0.9 | 14.08 | 5.63 | 99.6 ± 1.3 | 99.64 ± 0.07 | 99.1 ± 0.3 |
| C-22 | 45 | 0.30 | 19.6 |  |  | 6.8 | 13.82 ± 0.23 | 181.3 ± 8.9 | 4.05 ± 0.37 | 21.6 ± 1.1 | 12.69 | 5.08 | 98.7 ± 1.7 | 99.55 ± 0.08 | 98.4 ± 0.4 |

***Supplementary Table S6. Complete effects of critical process parameters on particle size, distribution width, and morphology (numerical source data for Figure 3)***

| **System** | **Parameter change** | **(Δ** $D₅₀$**) (µm; 95% CI)** | **p** | **Δ PSD span (95% CI)** | **p** | **Δ aspect ratio (95% CI)** | **p** |
| --- | --- | --- | --- | --- | --- | --- | --- |
| Paracetamol | $\tau$: 15→45 min | +87.0 (62.4 to 111.6) | <0.001 | −0.300 (−0.404 to −0.196) | <0.001 | −0.10 (−0.20 to −0.01) | 0.041 |
|  | $\sigma$: 0.10→0.30 | +43.0 (22.0 to 64.0) | 0.002 | +0.180 (0.054 to 0.306) | 0.008 | +0.08 (−0.01 to 0.17) | 0.078 |
|  | Seed: 0.25→1.25% | +38.0 (15.7 to 60.3) | 0.004 | −0.240 (−0.358 to −0.122) | 0.001 | −0.08 (−0.16 to 0.00) | 0.056 |
|  | $P/V$: 0.20→0.70 W L⁻¹ | −18.0 (−35.4 to −0.6) | 0.043 | −0.230 (−0.350 to −0.110) | 0.002 | −0.21 (−0.31 to −0.11) | <0.001 |
| Carbamazepine | $\tau$: 15→45 min | +59.0 (41.2 to 76.8) | <0.001 | −0.300 (−0.421 to −0.179) | <0.001 | −1.29 (−1.70 to −0.88) |  |
|  | $\sigma$: 0.10→0.30 | +25.0 (9.1 to 40.9) | 0.005 | +0.350 (0.214 to 0.486) |  | +1.17 (0.76 to 1.58) |  |
|  | Seed: 0.25→1.25% | +32.0 (14.2 to 49.8) | 0.002 | −0.270 (−0.396 to −0.144) | 0.001 | −0.67 (−1.02 to −0.32) | 0.002 |
|  | $P/V$: 0.20→0.70 W L⁻¹ | −13.0 (−25.5 to −0.5) | 0.042 | −0.380 (−0.510 to −0.250) | <0.001 | −1.27 (−1.65 to −0.89) | <0.001 |

***Supplementary Table S7. Complete effects of critical process parameters on chemical purity, polymorphic purity, recovery, and space-time yield (numerical source data for Figure 3)***

| **System** | **Parameter change** | **Δ chemical purity (percentage points)** | **p** | **Δ target polymorph (percentage points)** | **p** | **Δ recovery (percentage points)** | **p** | **Δ** $STY$ **(g L⁻¹ h⁻¹)** | **p** |
| --- | --- | --- | --- | --- | --- | --- | --- | --- | --- |
| Paracetamol | $\tau$: 15→45 min | +0.03 (−0.02 to 0.08) | 0.220 | +0.18 (0.03 to 0.33) | 0.022 | +5.2 (3.6 to 6.8) | <0.001 | −5.8 (−7.0 to −4.6) | <0.001 |
|  | $\sigma$: 0.10→0.30 | −0.07 (−0.12 to −0.02) | 0.009 | −0.34 (−0.54 to −0.14) | 0.002 | +12.0 (10.3 to 13.7) |  | +9.2 (7.6 to 10.8) |  |
|  | Seed: 0.25→1.25% | +0.02 (−0.03 to 0.07) | 0.402 | +0.40 (0.21 to 0.59) | <0.001 | +1.6 (0.5 to 2.7) | 0.008 | +1.2 (0.3 to 2.1) | 0.012 |
|  | $P/V$: 0.20→0.70 W L⁻¹ | +0.01 (−0.04 to 0.06) | 0.681 | +0.20 (0.03 to 0.37) | 0.024 | +0.8 (0.1 to 1.5) | 0.031 | +0.7 (0.1 to 1.3) | 0.026 |
| Carbamazepine | $\tau$: 15→45 min | +0.02 (−0.04 to 0.08) | 0.486 | +0.62 (0.31 to 0.93) | 0.001 | +4.8 (3.3 to 6.3) | <0.001 | −2.3 (−3.1 to −1.5) | <0.001 |
|  | $\sigma$: 0.10→0.30 | −0.09 (−0.15 to −0.03) | 0.006 | −1.16 (−1.49 to −0.83) | <0.001 | +11.4 (9.8 to 13.0) |  | +5.7 (4.7 to 6.7) |  |
|  | Seed: 0.25→1.25% | +0.03 (−0.03 to 0.09) | 0.317 | +1.22 (0.88 to 1.56) |  | +2.0 (0.8 to 3.2) | 0.003 | +1.1 (0.3 to 1.9) | 0.010 |
|  | $P/V$: 0.20→0.70 W L⁻¹ | +0.02 (−0.04 to 0.08) | 0.512 | +0.58 (0.26 to 0.90) | 0.002 | +1.1 (0.2 to 2.0) | 0.020 | +0.8 (0.2 to 1.4) | 0.013 |

***Supplementary Table S8. Adequacy statistics for the second-order response-surface models***

| **System** | **Response** | $R^{2}$ | **Adjusted** $R^{2}$ | **Predicted** $R^{2}$ | **RMSE** | **CV (%)** | **Model p** | **Lack-of-fit p** | **Maximum VIF** |
| --- | --- | --- | --- | --- | --- | --- | --- | --- | --- |
| Paracetamol | $D₅₀$ (µm) | 0.948 | 0.896 | 0.811 | 14.6 | 7.1 | <0.001 | 0.184 | 2.13 |
|  | PSD span | 0.925 | 0.850 | 0.742 | 0.071 | 5.0 |  | 0.236 |  |
|  | Aspect ratio | 0.906 | 0.812 | 0.701 | 0.052 | 3.4 |  | 0.317 |  |
|  | Chemical purity (%) | 0.882 | 0.764 | 0.612 | 0.060 | 0.06 | 0.003 | 0.412 |  |
|  | Form I (%) | 0.921 | 0.842 | 0.721 | 0.170 | 0.17 | <0.001 | 0.268 |  |
|  | Recovery (%) | 0.962 | 0.924 | 0.861 | 0.82 | 5.1 |  | 0.301 |  |
|  | $STY$ (g L⁻¹ h⁻¹) | 0.953 | 0.906 | 0.832 | 0.73 | 4.5 |  | 0.214 |  |
| Carbamazepine | $D₅₀$ (µm) | 0.941 | 0.882 | 0.798 | 9.1 | 6.9 |  | 0.192 | 2.18 |
|  | PSD span | 0.936 | 0.872 | 0.781 | 0.082 | 4.8 |  | 0.247 |  |
|  | Aspect ratio | 0.955 | 0.910 | 0.844 | 0.210 | 4.6 |  | 0.306 |  |
|  | Chemical purity (%) | 0.874 | 0.748 | 0.603 | 0.071 | 0.07 | 0.004 | 0.391 |  |
|  | Form III (%) | 0.958 | 0.916 | 0.853 | 0.280 | 0.29 | <0.001 | 0.288 |  |
|  | Recovery (%) | 0.967 | 0.934 | 0.879 | 0.76 | 4.8 |  | 0.265 |  |
|  | $STY$ (g L⁻¹ h⁻¹) | 0.946 | 0.892 | 0.817 | 0.36 | 6.1 |  | 0.322 |  |

***Supplementary Table S9. Significant quadratic and interaction terms retained in the response-surface models***

| **System** | **Response** | **Model term** | **Coded coefficient** | **Standard error** | **p** |
| --- | --- | --- | --- | --- | --- |
| Paracetamol | $D₅₀$ | $A^{2}$ | −10.20 | 4.30 | 0.032 |
|  |  | $(BD)$ | −8.10 | 3.70 | 0.047 |
|  | PSD span | $B^{2}$ | +0.080 | 0.031 | 0.021 |
|  |  | $(AD)$ | −0.061 | 0.026 | 0.035 |
|  | Recovery | $A^{2}$ | −0.55 | 0.21 | 0.019 |
|  |  | $B^{2}$ | −0.72 | 0.22 | 0.006 |
|  |  | $(AB)$ | +0.64 | 0.23 | 0.014 |
|  | $STY$ | $A^{2}$ | −0.48 | 0.19 | 0.024 |
|  |  | $(AB)$ | −0.81 | 0.25 | 0.006 |
|  | Form I | $(BC)$ | +0.16 | 0.06 | 0.018 |

***Supplementary Table S9 (continued). Significant quadratic and interaction terms retained in the response-surface models***

| **System** | **Response** | **Model term** | **Coded coefficient** | **Standard error** | **p** |
| --- | --- | --- | --- | --- | --- |
| Carbamazepine | $D₅₀$ | $A^{2}$ | −7.80 | 3.10 | 0.024 |
|  |  | $(BD)$ | −5.90 | 2.40 | 0.027 |
|  | PSD span | $B^{2}$ | +0.110 | 0.036 | 0.009 |
|  |  | $(BD)$ | −0.083 | 0.030 | 0.014 |
|  | Aspect ratio | $B^{2}$ | +0.310 | 0.090 | 0.004 |
|  |  | $(BD)$ | −0.280 | 0.095 | 0.011 |
|  |  | $(CD)$ | −0.220 | 0.088 | 0.025 |
|  | Recovery | $A^{2}$ | −0.61 | 0.20 | 0.009 |
|  |  | $B^{2}$ | −0.78 | 0.23 | 0.004 |
|  |  | $(AB)$ | +0.59 | 0.21 | 0.013 |
|  | $STY$ |  | −0.44 | 0.16 | 0.015 |
|  | Form III | $B^{2}$ | −0.43 | 0.13 | 0.005 |
|  |  | $(BC)$ | +0.37 | 0.12 | 0.008 |
|  |  | $(BD)$ | +0.28 | 0.11 | 0.022 |

***Note.*** *(A=*$\tau$*), (B=*$\sigma$*), (C=*$\varphi\_seed$*), and (D=*$P/V$*).*

***Supplementary Table S10. Unabridged optimized operating conditions and experimental confirmation***

| **System and condition** | $\tau$ **(min)** | $\sigma$ | **Seed (%)** | $P/V$ **(W L⁻¹)** | **Water (% v/v)** | $D₅₀$**: predicted / observed / error** | **Span: predicted / observed / error** | **Aspect ratio: predicted / observed / error** | **Chemical purity (%): predicted / observed** | **Target polymorph (%): predicted / observed** | **Recovery (%): predicted / observed / error** | $STY$**: predicted / observed / error** | **Fouling rate: predicted / observed** | **Desirability: predicted / observed** |
| --- | --- | --- | --- | --- | --- | --- | --- | --- | --- | --- | --- | --- | --- | --- |
| Paracetamol optimum | 33.6 | 0.261 | 1.08 | 0.61 | — | 252.4 / 248.6 ± 6.8 / −1.5% | 1.20 / 1.23 ± 0.03 / +2.5% | 1.47 / 1.49 ± 0.05 / +1.4% | 99.74 / 99.72 ± 0.04 | 99.6 / 99.5 ± 0.2 | 19.7 / 19.4 ± 0.7 / −1.5% | 16.72 / 16.46 ± 0.59 / −1.6% | 0.62 / 0.66 ± 0.08 | 0.914 / 0.899 |
| Paracetamol robustness | 29.4 | 0.292 | 1.15 | 0.66 | — | 245.2 / 231.8 ± 7.4 / −5.5% | 1.28 / 1.31 ± 0.04 / +2.3% | 1.47 / 1.52 ± 0.06 / +3.4% | 99.73 / 99.68 ± 0.05 | 99.6 / 99.1 ± 0.3 | 20.6 / 20.9 ± 0.8 / +1.5% | 19.29 / 20.27 ± 0.78 / +5.1% | 0.88 / 0.94 ± 0.10 | 0.842 / 0.819 |
| Carbamazepine optimum | 36.8 | 0.253 | 1.18 | 0.64 | 17.1 | 171.6 / 168.2 ± 5.9 / −2.0% | 1.38 / 1.42 ± 0.04 / +2.9% | 3.58 / 3.69 ± 0.16 / +3.1% | 99.65 / 99.62 ± 0.05 | 99.3 / 99.1 ± 0.2 | 19.1 / 18.8 ± 0.7 / −1.6% | 5.72 / 5.63 ± 0.21 / −1.6% | 0.78 / 0.83 ± 0.09 | 0.901 / 0.883 |
| Carbamazepine robustness | 31.2 | 0.291 | 1.25 | 0.70 | 19.2 | 164.6 / 157.7 ± 6.4 / −4.2% | 1.46 / 1.52 ± 0.05 / +4.4% | 3.80 / 4.03 ± 0.19 / +6.2% | 99.63 / 99.55 ± 0.06 | 99.2 / 98.3 ± 0.3 | 20.2 / 21.1 ± 0.8 / +4.3% | 7.49 / 7.19 ± 0.27 / −4.0% | 1.10 / 1.18 ± 0.12 | 0.821 / 0.796 |

***Note.*** *Observed values are mean ± SD from three independent confirmation runs.* $STY$ *is expressed as g L⁻¹ h⁻¹, and fouling rate as g m⁻² h⁻¹.*

***Supplementary Table S11. Complete calibration, internal-validation, and external-validation results for the population-balance models (numerical source data for Figure 5)***

| **System** | **Dataset** | **n** | **Output** | **RMSE** | **NRMSE (%)** | **MAPE (%)** | $R^{2}$ | **Mean bias** |
| --- | --- | --- | --- | --- | --- | --- | --- | --- |
| Paracetamol | Calibration | 22 | $D₅₀$ (µm) | 10.8 | 4.2 | 3.7 | 0.957 | −1.2 µm |
|  |  |  | $D₉₀$ (µm) | 21.6 | 4.8 | 4.1 | 0.948 | −3.7 µm |
|  |  |  | Dissolved concentration (g L⁻¹) | 0.48 | 1.2 | 1.0 | 0.972 | +0.06 g L⁻¹ |
|  |  |  | Crystal flow (mg min⁻¹) | 1.15 | 3.1 | 2.7 | 0.961 | −0.18 mg min⁻¹ |
|  | Internal validation | 7 | $D₅₀$ (µm) | 14.2 | 5.5 | 4.8 | 0.932 | +2.3 µm |
|  |  |  | $D₉₀$ (µm) | 28.4 | 6.3 | 5.6 | 0.918 | +5.1 µm |
|  |  |  | Dissolved concentration (g L⁻¹) | 0.67 | 1.7 | 1.5 | 0.951 | −0.11 g L⁻¹ |
|  |  |  | Crystal flow (mg min⁻¹) | 1.62 | 4.4 | 3.9 | 0.936 | +0.29 mg min⁻¹ |
|  | External validation | 9 | $D₅₀$ (µm) | 16.1 | 6.2 | 5.2 | 0.919 | −3.0 µm |
|  |  |  | $D₉₀$ (µm) | 31.7 | 7.0 | 6.1 | 0.902 | −6.4 µm |
|  |  |  | Dissolved concentration (g L⁻¹) | 0.73 | 1.8 | 1.6 | 0.943 | +0.14 g L⁻¹ |
|  |  |  | Crystal flow (mg min⁻¹) | 1.78 | 4.8 | 4.2 | 0.925 | −0.34 mg min⁻¹ |

***Supplementary Table S11 (continued). Complete calibration, internal-validation, and external-validation results for the population-balance models (numerical source data for Figure 5)***

| **System** | **Dataset** | **n** | **Output** | **RMSE** | **NRMSE (%)** | **MAPE (%)** | $R^{2}$ | **Mean bias** |
| --- | --- | --- | --- | --- | --- | --- | --- | --- |
| Carbamazepine | Calibration | 22 | $D₅₀$ (µm) | 7.4 | 5.3 | 4.5 | 0.951 | −0.8 µm |
|  |  |  | $D₉₀$ (µm) | 15.8 | 5.7 | 4.9 | 0.943 | −2.6 µm |
|  |  |  | Dissolved concentration (g L⁻¹) | 0.31 | 1.9 | 1.6 | 0.968 | +0.04 g L⁻¹ |
|  |  |  | Crystal flow (mg min⁻¹) | 0.54 | 3.8 | 3.2 | 0.956 | −0.09 mg min⁻¹ |
|  | Internal validation | 7 | $D₅₀$ (µm) | 9.8 | 7.0 | 6.0 | 0.925 | +1.7 µm |
|  |  |  | $D₉₀$ (µm) | 21.3 | 7.7 | 6.7 | 0.909 | +3.8 µm |
|  |  |  | Dissolved concentration (g L⁻¹) | 0.43 | 2.7 | 2.2 | 0.947 | −0.08 g L⁻¹ |
|  |  |  | Crystal flow (mg min⁻¹) | 0.72 | 5.1 | 4.4 | 0.931 | +0.14 mg min⁻¹ |
|  | External validation | 9 | $D₅₀$ (µm) | 11.2 | 7.9 | 6.8 | 0.911 | −2.1 µm |
|  |  |  | $D₉₀$ (µm) | 24.6 | 8.8 | 7.5 | 0.894 | −4.5 µm |
|  |  |  | Dissolved concentration (g L⁻¹) | 0.49 | 3.0 | 2.6 | 0.936 | +0.10 g L⁻¹ |
|  |  |  | Crystal flow (mg min⁻¹) | 0.81 | 5.7 | 4.9 | 0.918 | −0.17 mg min⁻¹ |

***Supplementary Table S12. Complete comparison of matched single-stage and two-stage MSMPR configurations (numerical source data for Figure 6)***

| **System** | **Response** | **Single-stage, mean ± SD** | **Two-stage, mean ± SD** | **Absolute difference** | **Relative change (%)** | **Holm-adjusted p** |
| --- | --- | --- | --- | --- | --- | --- |
| Paracetamol | $D₁₀$ (µm) | 88.5 ± 4.1 | 111.2 ± 4.8 | +22.7 | +25.6 | 0.003 |
|  | $D₅₀$ (µm) | 248.6 ± 6.8 | 287.9 ± 8.1 | +39.3 | +15.8 | 0.002 |
|  | $D₉₀$ (µm) | 394.3 ± 12.7 | 413.5 ± 13.4 | +19.2 | +4.9 | 0.041 |
|  | PSD span | 1.23 ± 0.03 | 1.05 ± 0.03 | −0.18 | −14.6 | 0.002 |
|  | Aspect ratio | 1.49 ± 0.05 | 1.44 ± 0.04 | −0.05 | −3.4 | 0.118 |
|  | Recovery (%) | 19.4 ± 0.7 | 20.1 ± 0.6 | +0.7 pp | +3.6 | 0.046 |
|  | $STY$ (g L⁻¹ h⁻¹) | 16.46 ± 0.59 | 17.05 ± 0.51 | +0.59 |  | 0.047 |
|  | Chemical purity (%) | 99.72 ± 0.04 | 99.74 ± 0.04 | +0.02 pp | +0.02 | 0.531 |
|  | Form I (%) | 99.5 ± 0.2 | 99.7 ± 0.1 | +0.2 pp | +0.2 | 0.093 |
|  | Mass-balance closure (%) | 99.4 ± 1.0 | 99.7 ± 0.8 | +0.3 pp | +0.3 | 0.604 |
|  | Fouling rate (g m⁻² h⁻¹) | 0.66 ± 0.08 | 0.38 ± 0.06 | −0.28 | −42.4 | 0.003 |
|  | Filtration time per 100 mL (s) | 42.6 ± 3.2 | 31.4 ± 2.7 | −11.2 | −26.3 | 0.004 |

***Supplementary Table S12 (continued). Complete comparison of matched single-stage and two-stage MSMPR configurations (numerical source data for Figure 6)***

| **System** | **Response** | **Single-stage, mean ± SD** | **Two-stage, mean ± SD** | **Absolute difference** | **Relative change (%)** | **Holm-adjusted p** |
| --- | --- | --- | --- | --- | --- | --- |
| Carbamazepine | $D₁₀$ (µm) | 58.1 ± 3.5 | 78.5 ± 4.0 | +20.4 | +35.1 | 0.002 |
|  | $D₅₀$ (µm) | 168.2 ± 5.9 | 191.0 ± 6.4 | +22.8 | +13.6 | 0.004 |
|  | $D₉₀$ (µm) | 296.9 ± 10.8 | 303.9 ± 11.2 | +7.0 | +2.4 | 0.214 |
|  | PSD span | 1.42 ± 0.04 | 1.18 ± 0.04 | −0.24 | −16.9 | 0.002 |
|  | Aspect ratio | 3.69 ± 0.16 | 3.05 ± 0.14 | −0.64 | −17.3 | 0.003 |
|  | Recovery (%) | 18.8 ± 0.7 | 19.7 ± 0.6 | +0.9 pp | +4.8 | 0.031 |
|  | $STY$ (g L⁻¹ h⁻¹) | 5.63 ± 0.21 | 5.90 ± 0.18 | +0.27 |  | 0.033 |
|  | Chemical purity (%) | 99.62 ± 0.05 | 99.65 ± 0.04 | +0.03 pp | +0.03 | 0.372 |
|  | Form III (%) | 99.1 ± 0.2 | 99.6 ± 0.2 | +0.5 pp | +0.5 | 0.012 |
|  | Mass-balance closure (%) | 99.2 ± 1.1 | 99.6 ± 0.9 | +0.4 pp | +0.4 | 0.487 |
|  | Fouling rate (g m⁻² h⁻¹) | 0.83 ± 0.09 | 0.45 ± 0.07 | −0.38 | −45.8 | 0.002 |
|  | Filtration time per 100 mL (s) | 96.8 ± 6.4 | 63.5 ± 5.1 | −33.3 | −34.4 |  |

***Supplementary Table S13. Unabridged fouling, filtration, and extended operational robustness at the optimized conditions***

| **System** | **Configuration** | **Extended duration (h)** | **Additional residence times** | **Deposit mass (mg)** | **Fouling rate (g m⁻² h⁻¹)** | **Product loss to deposits (%)** | **Filtration time per 100 mL (s)** | **Specific cake resistance (×10¹⁰ m kg⁻¹)** | **Cake moisture (%)** | **Outlet-flow CV (%)** | **Maximum flow deviation (%)** | $D₅₀$ **CV (%)** | **Concentration CV (%)** | **Minimum target polymorph (%)** | **Operational interruptions** |
| --- | --- | --- | --- | --- | --- | --- | --- | --- | --- | --- | --- | --- | --- | --- | --- |
| Paracetamol | Single-stage | 6.72 | 12 | 159.7 ± 18.6 | 0.66 ± 0.08 | 0.96 | 42.6 ± 3.2 | 1.82 ± 0.16 | 20.8 ± 1.3 | 1.1 | 2.8 | 2.8 | 1.9 | 99.3 | 0 |
|  | Two-stage |  |  | 109.8 ± 15.4 | 0.38 ± 0.06 | 0.64 | 31.4 ± 2.7 | 1.24 ± 0.12 | 18.9 ± 1.1 | 0.8 | 2.0 | 2.1 | 1.5 | 99.5 |  |
| Carbamazepine | Single-stage | 7.36 |  | 219.9 ± 23.8 | 0.83 ± 0.09 | 3.54 | 96.8 ± 6.4 | 4.76 ± 0.35 | 31.4 ± 1.8 | 1.6 | 3.9 | 3.5 | 2.6 | 98.8 |  |
|  | Two-stage |  |  | 142.4 ± 19.6 | 0.45 ± 0.07 | 2.19 | 63.5 ± 5.1 | 2.98 ± 0.27 | 26.7 ± 1.5 | 1.0 | 2.5 | 2.4 | 1.8 | 99.3 |  |

## Additional response-surface methodology documentation

The same 29-run Box–Behnken matrix was executed independently for paracetamol and carbamazepine; Supplementary Table S14 reports standard design order, while actual execution was randomized within experimental-day blocks.

***Supplementary Table S14. Four-factor Box–Behnken design matrix used independently for each API***

| **Design run** | **A** | **B** | **C** | **D** | $\tau$ (min) | $\sigma$ | **Seed (%)** | $P/V$ (W L⁻¹) |
| --- | --- | --- | --- | --- | --- | --- | --- | --- |
| 1 | -1 | -1 | 0 | 0 | 15 | 0.10 | 0.75 | 0.45 |
| 2 |  | 1 |  |  |  | 0.30 |  |  |
| 3 | 1 | -1 |  |  | 45 | 0.10 |  |  |
| 4 |  | 1 |  |  |  | 0.30 |  |  |
| 5 | -1 | 0 | -1 |  | 15 | 0.20 | 0.25 |  |
| 6 |  |  | 1 |  |  |  | 1.25 |  |
| 7 | 1 |  | -1 |  | 45 |  | 0.25 |  |
| 8 |  |  | 1 |  |  |  | 1.25 |  |
| 9 | -1 |  | 0 | -1 | 15 |  | 0.75 | 0.20 |
| 10 |  |  |  | 1 |  |  |  | 0.70 |
| 11 | 1 |  |  | -1 | 45 |  |  | 0.20 |
| 12 |  |  |  | 1 |  |  |  | 0.70 |
| 13 | 0 | -1 | -1 | 0 | 30 | 0.10 | 0.25 | 0.45 |
| 14 |  |  | 1 |  |  |  | 1.25 |  |
| 15 |  | 1 | -1 |  |  | 0.30 | 0.25 |  |
| 16 |  |  | 1 |  |  |  | 1.25 |  |
| 17 |  | -1 | 0 | -1 |  | 0.10 | 0.75 | 0.20 |
| 18 |  |  |  | 1 |  |  |  | 0.70 |
| 19 |  | 1 |  | -1 |  | 0.30 |  | 0.20 |
| 20 |  |  |  | 1 |  |  |  | 0.70 |
| 21 |  | 0 | -1 | -1 |  | 0.20 | 0.25 | 0.20 |
| 22 |  |  |  | 1 |  |  |  | 0.70 |
| 23 |  |  | 1 | -1 |  |  | 1.25 | 0.20 |
| 24 |  |  |  | 1 |  |  |  | 0.70 |
| 25 |  |  | 0 | 0 |  |  | 0.75 | 0.45 |
| 26 |  |  |  |  |  |  |  |  |
| 27 |  |  |  |  |  |  |  |  |
| 28 |  |  |  |  |  |  |  |  |
| 29 |  |  |  |  |  |  |  |  |

The response-level ANOVA components are reported in Supplementary Table S15. Model degrees of freedom were 14, residual degrees of freedom were 14, and the five center points provided four pure-error degrees of freedom for lack-of-fit testing.

***Supplementary Table S15. Detailed analysis of variance for the second-order response-surface models***

| **System** | **Response** | **Model F** | **Model p** | **LOF F** | **LOF p** |
| --- | --- | --- | --- | --- | --- |
| Paracetamol | $D₅₀$ | 18.23 | <0.001 | 2.43 | 0.184 |
|  | PSD span | 12.33 |  | 1.82 | 0.236 |
|  | Aspect ratio | 9.64 |  | 1.38 | 0.317 |
|  | Chemical purity | 7.47 | 0.003 | 1.04 | 0.412 |
|  | Form I | 11.66 | <0.001 | 1.62 | 0.268 |
|  | Recovery | 25.31 |  | 1.47 | 0.301 |
|  | $STY$ | 20.28 |  | 2.07 | 0.214 |
| Carbamazepine | $D₅₀$ | 15.95 |  | 2.36 | 0.192 |
|  | PSD span | 14.63 |  | 1.77 | 0.247 |
|  | Aspect ratio | 21.22 |  | 1.42 | 0.306 |
|  | Chemical purity | 6.94 | 0.004 | 1.12 | 0.391 |
|  | Form III | 22.81 | <0.001 | 1.51 | 0.288 |
|  | Recovery | 29.3 |  | 1.64 | 0.265 |
|  | $STY$ | 17.52 |  | 1.36 | 0.322 |

Supplementary Table S16 summarizes the residual checks used with the diagnostic panels in Supplementary Figure S1. No model showed a studentized residual beyond ±3, Cook’s D ≥1, rejected residual normality, or rejected homoscedasticity.

***Supplementary Table S16. Regression and residual diagnostics for the fitted response-surface models***

| **System** | **Response** | **Shapiro–Wilk p** | **Breusch–Pagan p** | **Max \|studentized residual\|** | **Max Cook’s D** | **Mean confirmation error (%)** |
| --- | --- | --- | --- | --- | --- | --- |
| Paracetamol | $D₅₀$ | 0.451 | 0.238 | 2.11 | 0.31 | 0.082 |
|  | PSD span | 0.326 | 0.414 | 1.94 | 0.28 | -0.036 |
|  | Aspect ratio | 0.719 | 0.532 | 2.03 | 0.24 | 0.018 |
|  | Chemical purity | 0.118 | 0.771 | 2.21 | 0.35 | -0.006 |
|  | Form I | 0.603 | 0.295 | 1.87 | 0.22 | 0.012 |
|  | Recovery | 0.864 | 0.184 | 2.16 | 0.39 | -0.041 |
|  | $STY$ | 0.297 | 0.092 | 2.34 | 0.42 | 0.057 |
| Carbamazepine | $D₅₀$ | 0.543 | 0.328 | 2.08 | 0.33 | -0.071 |
|  | PSD span | 0.681 | 0.447 | 1.92 | 0.26 | 0.029 |
|  | Aspect ratio | 0.232 | 0.156 | 2.27 | 0.37 | -0.048 |
|  | Chemical purity | 0.391 | 0.684 | 1.89 | 0.21 | 0.004 |
|  | Form III | 0.756 | 0.273 | 2.06 | 0.29 | -0.015 |
|  | Recovery | 0.477 | 0.119 | 2.19 | 0.4 | 0.052 |
|  | $STY$ | 0.344 | 0.203 | 2.12 | 0.32 | -0.033 |

**Supplementary Equations**

Supplementary Equations S1–S14 give the fitted coded models. Coefficients are rounded for presentation; optimization and confirmation used unrounded coefficients.

Paracetamol $D₅₀$:

$$D₅₀ = 225.815 + 43.500A + 21.500B + 19.000C - 9.000D - 10.200A^{2} - 8.100BD$$

(S1)

Paracetamol PSD span:

$$Span = 1.314 - 0.150A + 0.090B - 0.120C - 0.115D + 0.080B^{2} - 0.061AD$$

(S2)

Paracetamol aspect ratio:

$$AR = 1.551 - 0.050A + 0.040B - 0.040C - 0.105D$$

(S3)

Paracetamol chemical purity:

$$Purity = 99.748 + 0.015A - 0.035B + 0.010C + 0.005D$$

(S4)

Paracetamol Form I:

$$Form I = 99.422 + 0.090A - 0.170B + 0.200C + 0.100D + 0.160BC$$

(S5)

Paracetamol recovery:

$$Recovery = 14.838 + 2.600A + 6.000B + 0.800C + 0.400D - 0.550A^{2} - 0.720B^{2} + 0.640AB$$

(S6)

Paracetamol $STY$:

$$STY = 14.136 - 2.900A + 4.600B + 0.600C + 0.350D - 0.480A^{2} - 0.810AB$$

(S7)

Carbamazepine $D₅₀$:

$$D₅₀ = 146.761 + 29.500A + 12.500B + 16.000C - 6.500D - 7.800A^{2} - 5.900BD$$

(S8)

Carbamazepine PSD span:

$$Span = 1.618 - 0.150A + 0.175B - 0.135C - 0.190D + 0.110B^{2} - 0.083BD$$

(S9)

Carbamazepine aspect ratio:

$$AR = 4.503 - 0.645A + 0.585B - 0.335C - 0.635D + 0.310B^{2} - 0.280BD - 0.220CD$$

(S10)

Carbamazepine chemical purity:

$$Purity = 99.649 + 0.010A - 0.045B + 0.015C + 0.010D$$

(S11)

Carbamazepine Form III:

$$Form III = 98.561 + 0.310A - 0.580B + 0.610C + 0.290D - 0.430B^{2} + 0.370BC + 0.280BD$$

(S12)

Carbamazepine recovery:

$$Recovery = 13.916 + 2.400A + 5.700B + 1.000C + 0.550D - 0.610A^{2} - 0.780B^{2} + 0.590AB$$

(S13)

Carbamazepine $STY$:

$$STY = 4.044 - 1.150A + 2.850B + 0.550C + 0.400D - 0.440A^{2}$$

(S14)

For all equations, $A$ is residence time, $B$ is relative supersaturation, $C$ is seed loading, and $D$ is power input per unit volume; all are coded from −1 to +1.

**Supplementary Figure**

Residual normality, constant variance, leverage, and influence were assessed for every response model. Supplementary Figure S1 shows one representative diagnostic set for the paracetamol median-particle-size model; the complete numerical diagnostics for all 14 fitted response models are reported in Supplementary Table S16.

***Supplementary Figure S1. Representative residual diagnostics for the paracetamol median-particle-size Box–Behnken response-surface model: normal-probability plot of externally studentized residuals, residuals versus predicted median particle size, residuals versus run order, and Cook’s-distance plot. Complete numerical diagnostics for all fitted response models are reported in Supplementary Table S16.***
